# Association between diet intake and trace elements concentrations in couples undergoing *in vitro* fertilization: a couple-based exploration

**DOI:** 10.3389/fnut.2026.1722802

**Published:** 2026-02-12

**Authors:** Caiyun Wu, Xuemei Wang, Lin Su, Xin Gao, Yaning Sun, Yanlan Tang, Wei Ju, Junjun Liu, Feng Ni, Hong Jiang

**Affiliations:** 1 Reproductive Medicine Center, Clinical College of Peopleʼs Liberation Army Affiliated to Anhui Medical University, Hefei, China; 2 Reproductive Medicine Center, the 901th Hospital of the Joint Logistics Support Force of Peopleʼs Liberation Army, Hefei, China; 3 School of Public Health, Anhui Medical University, Hefei, Anhui, China

**Keywords:** dietary, factor analysis, IVF, reproductive health, trace elements

## Abstract

**Objective:**

This study aimed to provide guidance for couples in dietary adjustments to optimize trace element concentrations for improved fertility and to offer a reference for formulating precise reproductive health policies.

**Design:**

In this prospective cohort study, we analyzed 1,066 couples undergoing *in vitro* fertilization (IVF) from 2020 to 2023. Dietary intake was assessed using a Food Frequency Questionnaire, and 21 trace elements were measured. Multiple linear regression and factor analysis were used to evaluate associations.

**Setting:**

Hefei City, Anhui Province, China.

**Participants:**

A total of 1,066 couples undergoing IVF treatment.

**Results:**

In this study, we found significant associations between dietary intake and trace element concentrations in couples undergoing *in vitro* fertilization (IVF). Results showed that red meat consumption was inversely associated with Thallium (Tl) but positively correlated with Tin (Sn) and Cerium (Ce). Moderate intake of animal offal and processed meats showed significant associations with Aluminum (Al). Moderate sugar-sweetened beverage consumption was inversely linked to Tl levels. Furthermore, the moderate consumption of pickled and fried foods, as well as coffee, exhibited positive correlations with the trace elements Al and Manganese (Mn). Notably, as tea consumption increased, levels of Cobalt (Co), Gallium (Ga), and Strontium (Sr) also exhibited a significant rise. Even after False Discovery Rate (FDR) correction, the positive associations between Al levels and the moderate consumption of animal offal, pickled/fried foods, and coffee remained robust. Additionally, whole grain intake demonstrated a significant positive association with Sn, while tea consumption remained positively correlated with Rubidium (Rb).

**Conclusion:**

Our study emphasizes the significant impact of dietary intake on trace metal exposure in infertile couples. These insights can guide future research and help couples optimize trace elements through dietary modifications.

## 1 Background

Globally, approximately 48.5 million couples of reproductive age were affected by infertility, defined clinically as the inability to conceive after 12 months of unprotected intercourse (1). In China, infertility among couples of reproductive age has become a serious public health issue, with the prevalence rate reaching 25% (2). With advancements in medicine, *in vitro* fertilization (IVF) and other assisted reproductive technologies (ART) have emerged as effective treatments for infertility. However, the success rates of IVF are influenced by various factors, including environmental and lifestyle factors. Recent studies have found that diet and concentrations of trace elements may significantly impact reproductive health, but the specific mechanisms and relationships in this field remain unclear (3–5).

Trace metal elements, such as zinc (Zn), selenium (Se), and manganese (Mn), are present at relatively low levels in the human body but serve important physiological functions (6). These elements are involved in various biochemical processes, including oxidative stress response, DNA synthesis, and hormone regulation (7, 8). Both excess and deficiency of trace metal elements could have adverse effects on reproductive health. Previous studies have found that lead (Pb), mercury (Hg), and cadmium (Cd) are associated with menstrual disorders, endometriosis, and spontaneous abortion in women (9, 10). Additionally, it has been confirmed that Zn and Se have positive effects on sperm formation and motility (11, 12).

Notably, diet represents a primary source of trace elements, and its influence should not be underestimated (13, 14). It plays a crucial role in the intake of trace elements, affecting not only their concentration in the body but also their bioavailability. Specifically, individuals may increase the levels of cadmium and selenium by eating vegetables and grains, as these elements are often abundant in the soil (13, 14). Additionally, essential trace elements such as Zn, magnesium (Mg), and molybdenum (Mo) can be obtained by consuming an appropriate amount of nuts, vegetables, grains, and legumes (14, 15). Furthermore, some metals, such as mercury and arsenic, are primarily associated with seafood, shellfish, and meat.

However, most studies primarily concentrated on certain common heavy metal, such as Pb, Hg, and Cd, thereby neglecting to comprehensively examine the effects of diet on the broader spectrum of metal elements (4, 16–19). Additionally, research on the association between trace metal elements and diet in relation to reproductive health mostly only focused on one partner of couples, overlooking the joint influence of both partners (4, 5, 18, 20). It is well known that couples living together typically share a similar environment, diet, and lifestyle. Furthermore, previous studies have confirmed that the diets of both partners can affect the levels of trace metal elements in bodies, potentially leading to reproductive system damage (21–23). Nevertheless, there is still a lack of research investigating the relationship between dietary patterns and trace element concentrations from a couple’s perspective.

To address this gap, we conducted a cohort study on the association between diet and 21 trace elements among 1,066 infertile couples undergoing IVF treatment, focusing on the perspectives of each and both partners. Our research aims to provide guidance for couples in dietary adjustments to reduce the negative impact of harmful elements on fertility and to offer a reference for formulating precise reproductive health policies.

## 2 Methodology

### 2.1 Population and study design

We conducted a prospective cohort study within the Infertility cohort from December 2020 to August 2023, which is a subset of the Reproductive Health of Childbearing Couples—Anhui Cohort (RHCC-AC). Couples participating in this study were those receiving ART treatment at the Reproductive Center of the Maternal and Child Health Care Hospital in Ma’anshan, as well as at the 901th Hospital of the Joint Logistics Support Force of People’s Liberation Army. The inclusion criteria for the study were as follows: (1) women aged 20 to 49 years and men aged 22 to 49 years; (2) couples diagnosed with infertility, defined as the inability to achieve clinical pregnancy after at least 1 year of unprotected intercourse; (3) indications for IVF, which included female factors like tubal issues or ovulation failure, as well as male factors or unexplained infertility. At enrollment, participants completed detailed questionnaires to gather information on their demographics, lifestyle, reproductive history, and medical background. Our study has been approved by the Ethics Committee of Anhui Medical University (No. 20189999), and all participants provided written informed consent.

Supplementary Figure S1 illustrates the flowchart of study participants included in our research. In total, 2,437 couples were enrolled in the cohort. Exclusions occurred for couples lacking electronic medical records (*n* = 799) or those who did not undergo ART treatment (*n* = 199). Additionally, couples with abnormal karyotypes (*n* = 73) or those experiencing abnormal fertilization, no oocytes retrieved, and no embryos available for transfer (*n* = 69) were also excluded. In addition, couples with missing diet data (*n* = 5) and couples who did not provide plasma samples for exposure measurements (*n* = 228) were removed from the analysis. Finally, 1,066 couples were included in the current analysis.

### 2.2 Trace elements measurements

Upon completion of the baseline and dietary questionnaires, venous blood was drawn from each partner’s cubital vein via anticoagulant collection tubes during their clinical visit. The blood was transferred into tubes containing anticoagulants and then centrifuged at 2,000 rpm for 10 min. Following centrifugation, the supernatant was collected and stored in 2 mL Eppendorf tubes at −80 °C until analysis.

To measure the concentrations of 21 trace elements in the plasma, we employed a direct dilution method, diluting 200 μL of plasma samples 20-fold with a solution containing 0.05% nitric acid, 0.05% Triton X-100, and 10 μg/L Au, utilizing an inductively coupled plasma mass spectrometer (ICP-MS) from Thermo Fisher Scientific. Each sample was measured in triplicate to ensure accuracy. Detection utilized the AccuStandard multi-element calibration solution (ICP-MS-CAL2-1, 10 μg/mL) and a multi-element internal standard solution (ICP-MS-200.8-IS-1, 10 μg/mL), with internal standards selected based on mass similarity principles. Most trace elements demonstrated a linear range of 0.01 to 20 ng/L, with the exception of iron (Fe), copper (Cu), Zn, and Mg. Calibration curves for all 21 elements showed linear correlation coefficients greater than 0.999. Recovery rates for the plasma metals ranged from 92.96 to 108.38%, and intra-day and inter-day precision varied from 1.67 to 11.67%. The limits of detection (LOD) for the trace elements and their corresponding internal standards are detailed in Supplementary Table S1. For analysis, concentrations below LOD were assigned values of LOD/√2. In subsequent statistical analyses, only trace metal elements detected in more than 50% of individual partners were included.

### 2.3 Dietary assessment

During the recruitment, we collected dietary information from participants utilizing a Food Frequency Questionnaire (FFQ). Study subjects were asked to recall their consumption frequency of 14 food items over the past month, including white rice, coarse grains, dark-colored vegetables, fruits, legumes and their products, nuts, red meat, organ meats, processed meats, sugary beverages, pickled or fried foods, coffee, tea, red wine, and beer. The frequency options were categorized as: never/rarely, once a month, 1–3 days per week, 4–6 days per week, and almost daily. In subsequent analyses, we reclassified the intake frequencies into “high intake” (4–6 days per week and almost daily), “low intake” (1–3 days per week, once a month, and never or rarely) to ensure a larger sample size for each category. In the couple-based analysis, participants were classified into three groups: the high intake group (where both partners demonstrated high intake), the moderate intake group (where one partner exhibited high intake), and the low intake group (where both partners exhibited low intake). Correspondingly, in the partner-specific models, each dietary item was treated as a binary variable (high vs. low intake) to facilitate the individual-level analysis.

### 2.4 Covariates

The determination of covariates was based on existing literature and their reasonable associations with trace elements (7, 18, 19, 24). In the couple-based model, we adjusted for several factors, including both partners’ age (continuous variable), residence area (central Anhui, southern Anhui and northern Anhui), personal annual income (<60,000 and ≥60,000 yuan per year), education level (less than high school, high school, and college and above), body mass index (BMI, continuous variable), smoking status (never, former or current), drinking status (never, frequent, and nearly every day), and sampling season (spring, summer, autumn, and winter). In the partner-specific model, we explored the relationship between each partner’s dietary status and the concentration of trace elements, while adjusting for each partner’s age (continuous variable), residence area (central Anhui, southern Anhui and northern Anhui), personal annual income (<60,000 and ≥60,000 yuan per year), education level (less than high school, high school, and college and above), BMI (continuous variable), smoking status (never, former or current), drinking status (never, frequent, and nearly every day), and sampling season (spring, summer, autumn, and winter).

### 2.5 Statistical analyses

The demographic and clinical characteristics of the study population were summarized using median (IQR: interquartile range) or mean (SD: standard deviation) for continuous variables, while categorical variables were expressed as frequency (proportion). For statistical analysis, Student’s t-test was applied to normally distributed variables, the Kruskal-Wallis H test was employed for skewed variables, and the Chi-square test was utilized for categorical variables. To enhance normality, natural logarithm transformation was performed on the concentrations of 21 trace metal elements in plasma. Additionally, Spearman correlation analysis was conducted to assess the relationships among trace metal elements within couples. Specifically, we used multiple linear regression models to investigate the relationships between 13 dietary items and 21 trace elements in couple-based and partner-specific models, respectively. The False Discovery Rate (FDR) approach was applied to adjust for multiple testing, with significance set at an adjusted *p*-value of < 0.05.

Given that the participants were individuals undergoing IVF treatment, red wine and beer were excluded from the analysis due to their low consumption frequency among the majority of participants. In the couple-based analysis, the mean concentrations of 21 trace elements for each couple were employed as the measure of couple-based exposure (25). To identify representative dietary patterns, we performed factor analysis based on the consumption frequencies of 13 food groups for couples and each individual partner. The suitability of the dietary data for factor analysis was verified using the Kaiser-Meyer-Olkin (KMO) measure of sampling adequacy (threshold > 0.6) and Bartlett’s test of sphericity (*p* < 0.05). Factors were extracted based on eigenvalues > 1.0, the scree plot, and the cumulative variance explained. A varimax orthogonal rotation was applied to enhance the interpretability of the factors and minimize the correlation between them. Food groups were considered to contribute significantly to a pattern if their absolute factor loadings were ≥ 0.40. The identified patterns were descriptively named according to the primary food groups within each factor. The complete factor loading matrix for all food groups is presented in Supplementary Tables S2–S4. Finally, factor scores were calculated using the regression method and subsequently entered into multiple linear regression models as independent variables to assess their associations with trace metal elements. All statistical analyses were performed using R version 4.3, with statistical significance defined at *p*-value < 0.05 two-tailed.

## 3 Results

### 3.1 Descriptive statistics

The characteristics of the 1,066 couples undergoing IVF treatment are presented in Table 1. Specifically, the mean (SD) age of females was 33.26 (5.21) years, while the mean age of the men was 33.90 (5.21) years. Among the participants, 44.0% of females were classified as overweight or obese, compared to 61.2% of males. Most females reported not actively smoking (94.5%) or consuming alcohol (80.0%) in the past 6 months, whereas the corresponding proportion for males were 65.3 and 37.4%, respectively. In addition, the distribution of participants living in central, northern, and southern Anhui was relatively balanced, of which 35.3, 30.9%, and 33.9% were female, and 35.5, 31.1%, and 33.4% were male, respectively.

**Table 1:** Population characteristics by parity status among couples (*n* = 1,066)^a^.

Characteristic	Female (*n* = 1,066)	Male (*n* = 1,066)
Age(years)	32.66 (5.21)	33.90 (5.94)
BMI (kg/m^2^)		
<18.5	55 (5.2)	34 (3.2)
18.5–23.9	542 (50.8)	380 (35.6)
24–27.9	321 (30.1)	409(38.4)
≥28	148 (13.9)	243 (22.8)
Education		
Less than high school	525 (49.2)	448 (42.0)
High school	179 (16.8)	220 (20.6)
College and above	362 (34.0)	398 (37.3)
Personal income (yuan/year)		
<60,000	832 (78.0)	516 (48.4)
≥60,000	234 (22.0)	550 (51.6)
Active smoking		
Never	1,007 (94.5)	450 (42.2)
Former or Current	59 (5.5)	616 (57.8)
Passive smoking		
Never	849 (79.6)	696 (65.3)
Frequent	42 (3.9)	85 (8.0)
Nearly every day	175 (16.4)	285 (26.7)
Alcohol use		
Never	853 (80.0)	399 (37.4)
Frequent	205 (19.2)	506 (47.5)
Nearly every day	8 (0.8)	161 (15.1)
Residence area		
Central Anhui	376 (35.3)	378 (35.5)
Northern Anhui	329 (30.9)	332 (31.1)
Southern Anhui	361 (33.9)	356 (33.4)

BMI, body mass index.

a Values are presented as mean (SD) for continuous variables or as n (%) for categorical variables.

**Figure 1:**
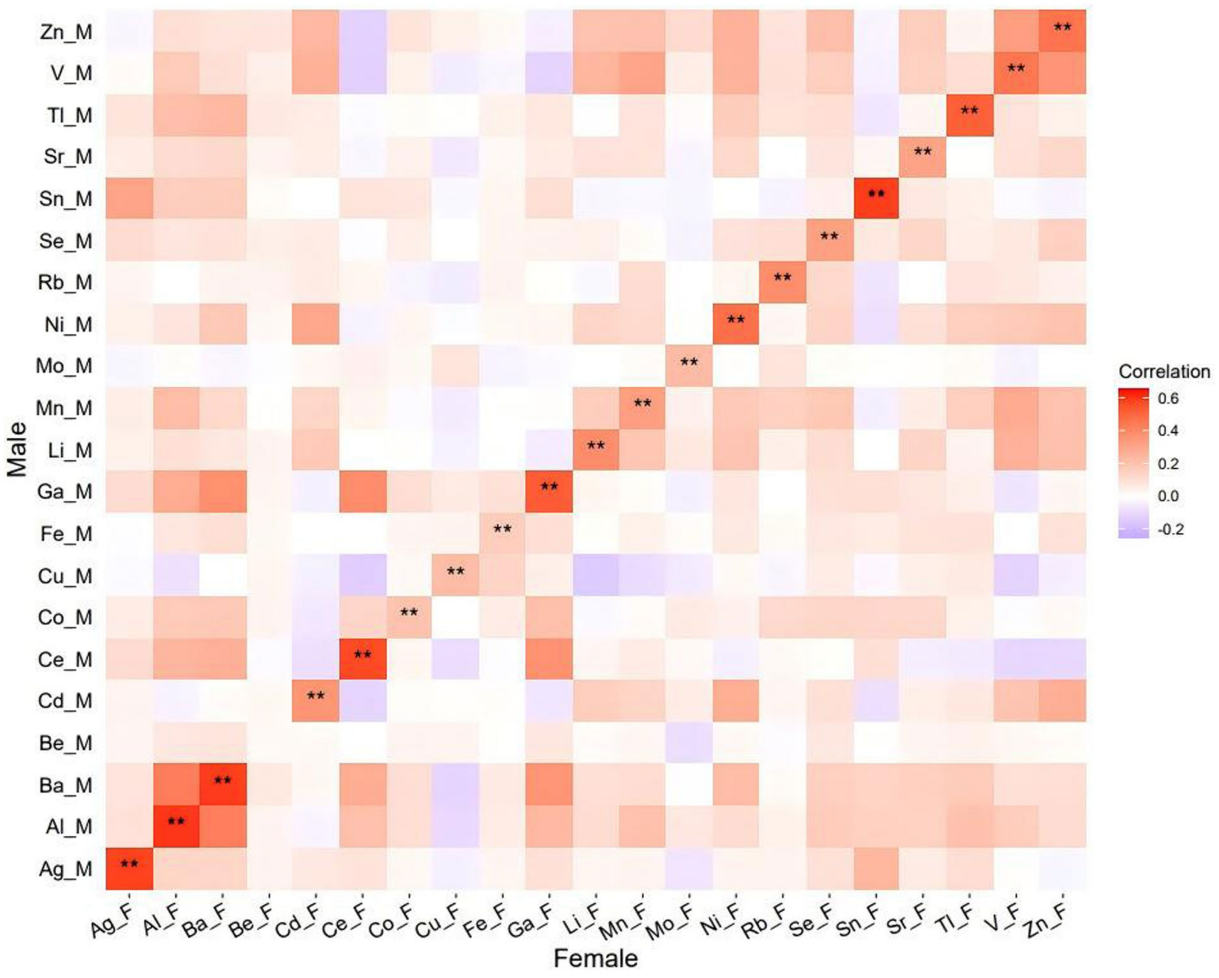
Spearman correlation matrix of trace elements within each partner. M and F represented the trace metal elements in males and females, respectively.

Among the 21 trace elements analyzed, beryllium (Be) was detected in 52.7% of females and 54.4% of males, while the majority of the other trace metal elements were found in over 90% of couples (Supplementary Table S1). The concentrations of individual trace elements varied, ranging from 0.05 to 3933.25 ng/mL in females and from 0.05 to 4338.89 ng/mL in males (Supplementary Table S1). Additionally, Spearman correlation analysis revealed significant correlations among most trace metals within couples (Figure 1).

### 3.2 Association between dietary intake and trace metal element concentrations from couples and each partner

In the couple-based analysis, after adjusting for shared confounding factors, moderate consumption of whole grains demonstrated a significant positive association with trace metal elements, including aluminum (Al), vanadium (V), manganese (Mn), tin (Sn), and silver (Ag), compared to low intake levels (Table 2). When one or both partners have high intake of dark-colored vegetables and fruits, levels of cerium (Ce) and gallium (Ga) significantly increased. Additionally, the high intake of legumes and soy products by both partners exhibited significant correlations with Al (95% CI: 0.00, 0.34) and molybdenum (Mo; 95% CI: 0.05, 0.38). Red meat consumption exhibited a negative association with thallium (Tl), while showing positive associations with Sn and Ce. Furthermore, we found significant associations between the moderate consumption of animal offal and processed meats with Al. The moderate intake of sugar-sweetened beverages was inversely associated with Tl levels. Moreover, the moderate consumption of pickled and fried foods, as well as coffee, exhibited positive correlations with trace metal elements Al and Mn. Importantly, as tea consumption increased, levels of cobalt (Co), gallium (Ga), and Sr. also exhibited a significant rise (Table 2). Notably, even after FDR correction, significant associations persisted between the moderate consumption of animal offal, pickled and fried foods, and coffee with Al levels. Furthermore, moderate intake of whole grains demonstrated a significant positive association with Sn, while tea consumption remained positively correlated with Rb (Supplementary Table S7).

**Table 2:** Association between dietary intake and trace elements concentrations among 1,066 couples.

Dietary intake	Element	Frequency of intake		
		Low intake	Moderate intake	High intake
White rice	Li	Reference	0.18(−0.06,0.43)	**0.25(0.00,0.49)**
	Ni	Reference	0.09(−0.16,0.35)	**0.27(0.01,0.52)**
Coarse grain	Al	Reference	**0.16(0.02,0.29)**	0.09(−0.17,0.34)
	V	Reference	**0.14(0.01,0.27)**	0.11(−0.13,0.36)
	Mn	Reference	**0.16(0.03,0.29)**	0.10(−0.15,0.35)
	Sn	Reference	**0.24(0.11,0.37)**	0.19(−0.06,0.44)
	Ag	Reference	**0.13(0.00,0.27)**	0.18(−0.08,0.43)
Dark-colored vegetables	Ga	Reference	**0.18(0.00,0.36)**	0.14(−0.04,0.32)
	Ce	Reference	**0.25(0.08,0.43)**	**0.20(0.02,0.38)**
Fruits	Ga	Reference	0.09(−0.06,0.24)	**0.23(0.07,0.39)**
	Ce	Reference	0.11(−0.04,0.26)	**0.24(0.08,0.40)**
Legumes and soy products	Al	Reference	0.04(−0.10,0.17)	**0.17(0.00,0.34)**
	Mo	Reference	0.03(−0.10,0.16)	**0.21(0.05,0.38)**
Nuts	Co	Reference	0.13(−0.01,0.26)	**0.24(0.01,0.48)**
Red meat	Sn	Reference	0.18(−0.01,0.36)	**0.20(0.02,0.39)**
	Ce	Reference	0.12(−0.06,0.30)	**0.24(0.06,0.42)**
	Se	Reference	0.04(−0.14,0.22)	0.18(0.00,0.37)
	Tl	Reference	**−0.15(−0.33,0.03)**	**−0.20(−0.38,-0.01)**
Animal offal	Al	Reference	**0.23(0.07,0.38)**	0.14(−0.19,0.48)
	Sr	Reference	**0.18(0.04,0.33)**	0.11(−0.20,0.43)
Processed meat	Al	Reference	**0.20(0.04,0.35)**	0.17(−0.20,0.54)
	Sr	Reference	0.11(−0.04,0.26)	−0.16(−0.52,0.19)
	Ba	Reference	0.07(−0.08,0.22)	**0.38(0.01,0.75)**
Sugar-sweetened beverages	Tl	Reference	**−0.16(−0.29,−0.02)**	0.05(−0.22,0.32)
Pickled or fried food	Al	Reference	**0.23(0.08,0.37)**	-0.02(−0.29,0.25)
	Mn	Reference	**0.19(0.05,0.32)**	−0.01(−0.27,0.25)
	Ni	Reference	**0.17(0.03,0.32)**	0.21(−0.06,0.47)
	Se	Reference	**−0.05(−0.18,0.09)**	**−0.27(−0.53,-0.00)**
Coffee	Al	Reference	**0.27(0.10,0.44)**	**0.47(0.04,0.91)**
	Mn	Reference	**0.19(0.02,0.36)**	0.31(−0.12,0.73)
	Co	Reference	**0.19(0.02,0.36)**	0.28(−0.15,0.71)
	Ni	Reference	**0.18(0.01,0.35)**	0.22(−0.21,0.66)
	Ba	Reference	0.16(−0.01,0.34)	**0.47(0.04,0.91)**
Tea	Fe	Reference	0.06(−0.07,0.19)	**0.27(0.06,0.48)**
	Mn	Reference	0.12(−0.01,0.25)	**0.23(0.03,0.44)**
	Co	Reference	**0.15(0.02,0.28)**	0.07(−0.14,0.28)
	Ni	Reference	0.11(−0.02,0.24)	**0.27(0.06,0.48)**
	Ga	Reference	0.10(−0.03,0.23)	**0.30(0.10,0.50)**
	Rb	Reference	0.10(−0.03,0.22)	**0.41(0.21,0.62)**
	Sr	Reference	**0.14(0.02,0.27)**	**0.24(0.04,0.44)**

To enhance readability and clarity, we have simplified the tables to highlight only the positive results. The bold font indicates statistically significant positive results (*p* < 0.05).

The partner-specific models showed that, in females before FDR correction, the high intake of coarse grains was positively associated with Sn level, but negatively associated with Cu and Se levels. Meanwhile the high intake of white rice was positively associated with Mo and Ni levels. Moreover we also find that the high intake of coke was negatively associated with Cu and Tl levels in female However, after the FDR correction, no association was found between dietary intake and the concentration of trace metal elements in the females (Supplementary Table S8). However, in males before FDR correction, the high intake of coffee, processed meats, pickled and fried foods, coke, as well as coarse grains, were significantly associated with Ag levels. Meanwhile the high intake of tea was positively associated with Rb level (Supplementary Table S9). Notably, after the FDR correction, the consumption of tea and coffee still showed a positive correlation with Rb and Ag levels in male (Supplementary Table S9).

### 3.3 Dietary pattern extraction

**Figure 2:**
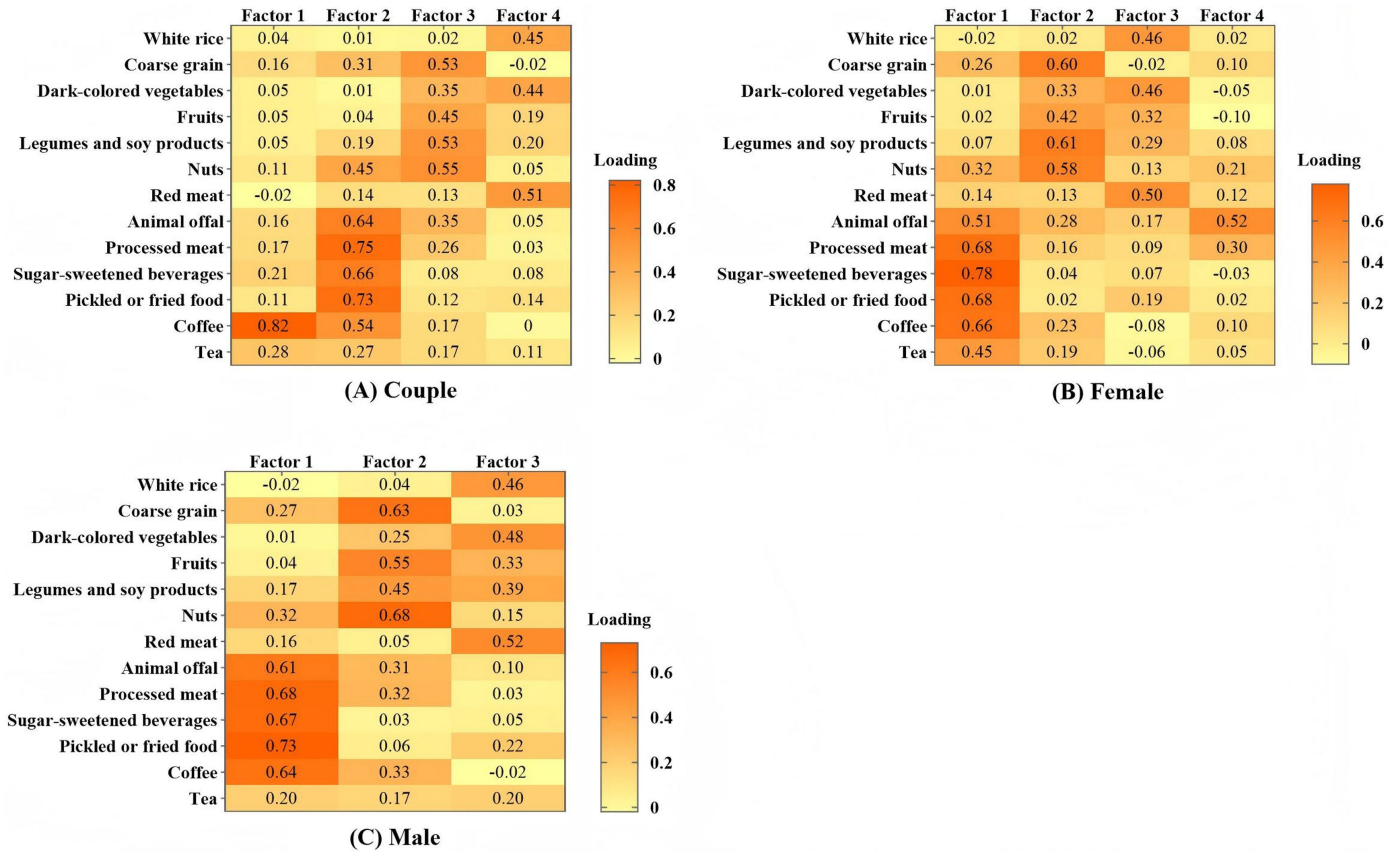
**(A)** The loading matrix of the determined common factors among 1,066 couples, which explained 45.0% of the total variance of the diet intake. **(B)** The loading matrix of the determined common factors among 1,066 females, which explained 43.0% of the total variance of the diet intake. **(C)** The loading matrix of the determined common factors among 1,066 males, which explained 41.0% of the total variance of the diet intake.

In the couples-based model, the factor analysis results showed that the KMO value was 0.88, and the Bartlett’s test of sphericity results were χ^2^ = 3949.99, *p* < 0.01. Furthermore, through factor analysis, we identified four dietary patterns, which collectively explained 45.0% of the variance across 13 dietary items. Factor 1, defined as the beverage dietary pattern, was mainly characterized by coffee and tea intake, with a factor loading of 0.28 for tea. Factor 2 called the processed dietary pattern, was characterized by higher factor loadings for nuts, animal offal, processed meats, sugar-sweetened beverages, preserved foods, and coffee. Factor 3 was the balanced dietary pattern, characterized by higher intakes of coarse grains, fruits, legumes and soy products, and nuts. Factor 4 was referred to as the traditional dietary pattern, with a primary focus on the consumption of white rice, vegetables, and red meat (Supplementary Table S4; Figure 2).

In the partner-specific model, the factor analysis results for females showed a KMO value of 0.85, and the Bartlett’s test of sphericity resulted in χ^2^ = 3638.20, *p* < 0.01. In females, four dietary patterns were determined through factor analysis, which explained 43.0% of the variance in 13 dietary items. Factor 1 was named the processed dietary pattern, characterized by higher intakes of animal offal, processed meats, sugary beverages, pickled foods, coffee, and tea. Factor 2 was referred to as the balanced dietary pattern, characterized by higher intakes of coarse grains, fruits, legumes and soy products, and nuts. Factor 3 was named the traditional dietary pattern, displaying higher factor loadings for white rice, vegetables, fruits, and red meat. Factor 4 was identified as the single animal offal dietary pattern, primarily characterized by high consumption of animal offal (Supplementary Table S2; Figure 2).

In males, the factor analysis results indicated a KMO value of 0.86, and the Bartlett’s test of sphericity showed χ^2^ = 3638.20, *p* < 0.01. Through factor analysis, we ultimately identified three dietary patterns, which accounted for 41.0% of the variance in 13 dietary items. Factor 1, characterized by high intakes of offal, processed meats, pickled foods, sugary beverages, and coffee, was named the processed dietary pattern. Factor 2 was termed the balanced dietary pattern, distinguished by higher consumption of coarse grains, fruits, legumes, and nuts. Factor 3 was identified as the traditional dietary pattern, characterized by higher intakes of white rice, vegetables, and red meat (Supplementary Table S3; Figure 2).

### 3.4 Association between dietary patterns and trace metal element concentrations from couples and each partner

In the couple-based model, higher adherence to the beverage dietary pattern was positively associated with the levels of Al, Ga, and Ba. Additionally, greater adherence to the processed dietary pattern was significantly related to higher Ni levels. The balanced dietary pattern with higher adherence, demonstrated a positive association with the levels of Ga, Sn, and Ce. Similarly, greater adherence to the traditional dietary pattern was positively associated with increased levels of Ni, Ce, and Mo. In contrast, higher adherence to the traditional dietary pattern was negatively associated with Sr. and Tl (Table 3).

**Table 3:** Association between dietary patterns and trace elements concentrations among 1,066 couples.

Element	Beverage dietary pattern			Processed dietary pattern			Balanced dietary pattern			Traditional dietary pattern		
	T1	T2	T3	T1	T2	T3	T1	T2	T3	T1	T2	T3
Be	Ref	0.10(−0.04,0.25)	0.11(−0.04,0.26)	Ref	0.09(−0.05,0.24)	0.02(−0.13,0.17)	Ref	0.03(−0.12,0.18)	0.02(−0.13,0.17)	Ref	0.07(−0.08,0.22)	−0.08(−0.24,0.07)
Al	Ref	−0.12(−0.27,0.03)	**0.15(0.00,0.30)**	Ref	−0.03(−0.18,0.11)	0.06(−0.08,0.21)	Ref	0.06(−0.09,0.20)	0.12(−0.03,0.27)	Ref	0.11(−0.04,0.26)	0.05(−0.10,0.21)
V	Ref	−0.12(−0.26,0.02)	−0.12(−0.26,0.02)	Ref	−0.00(−0.14,0.14)	0.10(−0.04,0.24)	Ref	−0.07(−0.21,0.07)	0.03(−0.11,0.17)	Ref	−0.01(−0.16,0.13)	−0.08(−0.23,0.06)
Li	Ref	−0.06(−0.20,0.09)	−0.10(−0.24,0.05)	Ref	−0.01(−0.15,0.14)	0.10(−0.04,0.25)	Ref	−0.02(−0.17,0.12)	0.08(−0.06,0.23)	Ref	0.03(−0.12,0.18)	−0.06(−0.21,0.09)
Fe	Ref	−0.08(−0.23,0.06)	0.07(−0.08,0.22)	Ref	−0.05(−0.19,0.10)	−0.03(−0.18,0.12)	Ref	−0.13(−0.27,0.02)	0.04(−0.11,0.19)	Ref	−0.06(−0.21,0.09)	−0.00(−0.15,0.15)
Mn	Ref	−0.14(−0.29,0.00)	0.06(−0.09,0.20)	Ref	−0.02(−0.16,0.12)	0.01(−0.14,0.15)	Ref	−0.11(−0.25,0.04)	0.07(−0.08,0.21)	Ref	0.04(−0.11,0.19)	0.11(−0.04,0.26)
Co	Ref	−0.09(−0.23,0.06)	0.05(−0.09,0.20)	Ref	0.09(−0.05,0.23)	0.10(−0.05,0.25)	Ref	−0.04(−0.19,0.11)	0.05(−0.10,0.19)	Ref	−0.05(−0.20,0.10)	−0.07(−0.22,0.08)
Ni	Ref	−0.05(−0.20,0.09)	0.09(−0.05,0.24)	Ref	0.14(−0.01,0.28)	**0.15(0.00,0.30)**	Ref	−0.05(−0.20,0.10)	0.06(−0.09,0.21)	Ref	**0.17(0.02,0.32)**	0.05(−0.10,0.20)
Ga	Ref	−0.03(−0.18,0.11)	**0.19(0.05,0.33)**	Ref	0.02(−0.12,0.16)	−0.06(−0.20,0.08)	Ref	**0.18(0.04,0.32)**	0.09(−0.06,0.23)	Ref	0.11(−0.04,0.26)	0.14(−0.01,0.29)
Rb	Ref	0.08(−0.07,0.22)	0.10(−0.05,0.24)	Ref	0.03(−0.11,0.18)	−0.01(−0.16,0.13)	Ref	0.01(−0.13,0.15)	0.01(−0.14,0.15)	Ref	0.06(−0.08,0.21)	0.12(−0.02,0.27)
Sn	Ref	−0.02(−0.16,0.13)	−0.04(−0.18,0.11)	Ref	0.06(−0.08,0.21)	0.04(−0.11,0.08)	Ref	**0.22(0.07,0.37)**	0.15(−0.00,0.30)	Ref	0.13(−0.02,0.28)	0.06(−0.09,0.21)
Sr	Ref	0.06(−0.21,0.08)	0.02(−0.12,0.17)	Ref	−0.02(−0.16,0.12)	0.06(−0.08,0.21)	Ref	0.03(−0.11,0.17)	0.11(−0.04,0.25)	Ref	**−0.17(−0.31,-0.02)**	−0.05(−0.20,0.10)
Ce	Ref	−0.06(−0.20,0.09)	0.13(−0.01,0.18)	Ref	0.04(−0.11,0.18)	−0.04(−0.18,0.11)	Ref	**0.15(0.01,0.30)**	0.07(−0.08,0.22)	Ref	**0.15(0.00,0.30)**	**0.18(0.03,0.33)**
Cu	Ref	0.04(−0.09,0.12)	0.02(−0.12,0.05)	Ref	−0.04(−0.18,0.09)	0.00(−0.14,0.14)	Ref	0.05(−0.09,0.19)	0.03(−0.11,0.17)	Ref	0.00(−0.14,0.15)	−0.08(−0.23,0.06)
Ag	Ref	−0.11(−0.26,0.03)	−0.01(−0.16,0.14)	Ref	0.02(−0.12,0.17)	0.08(−0.07,0.22)	Ref	0.11(−0.04,0.26)	0.05(−0.10,0.20)	Ref	0.02(−0.13,0.17)	0.01(−0.14,0.16)
Cd	Ref	−0.07(−0.22,0.08)	−0.01(−0.15,0.14)	Ref	0.11(−0.04,0.25)	0.06(−0.08,0.21)	Ref	0.01(−0.14,0.16)	0.07(−0.07,0.22)	Ref	0.13(−0.02,0.28)	−0.00(−0.15,0.15)
Ba	Ref	−0.11(−0.26,0.04)	**0.17(0.02,0.31)**	Ref	0.02(−0.12,0.17)	0.04(−0.11,0.19)	Ref	0.11(−0.04,0.26)	0.06(−0.09,0.21)	Ref	0.14(−0.01,0.29)	0.11(−0.04,0.26)
Se	Ref	0.05(−0.09,0.19)	0.08(−0.06,0.22)	Ref	−0.00(−0.14,0.14)	−0.08(−0.22,0.06)	Ref	0.10(−0.04,0.24)	−0.02(−0.17,0.12)	Ref	0.12(−0.03,0.27)	0.15(−0.00,0.30)
Zn	Ref	−0.07(−0.22,0.07)	−0.05(−0.19,0.10)	Ref	−0.02(−0.17,0.12)	0.04(−0.11,0.19)	Ref	0.01(−0.14,0.15)	0.10(−0.04,0.25)	Ref	0.04(−0.11,0.19)	0.05(−0.10,0.20)
Tl	Ref	−0.06(−0.21,0.08)	0.09(−0.05,0.24)	Ref	−0.06(−0.21,0.08)	−0.02(−0.16,0.13)	Ref	−0.07(−0.22,0.08)	−0.04(−0.19,0.11)	Ref	−0.06(−0.21,0.09)	**−0.17(−0.32,-0.02)**
Mo	Ref	0.01(−0.14,0.15)	0.05(−0.09,0.20)	Ref	−0.09(−0.24,0.05)	−0.03(−0.18,0.11)	Ref	−0.02(−0.17,0.12)	0.02(−0.13,0.17)	Ref	**0.16(0.01,0.31)**	**0.15(0.00,0.30)**

Ref, Reference. To enhance readability and clarity, we have simplified the tables to highlight only the positive results. The bold font indicates statistically significant positive results (*p* < 0.05).

In the partner-specific model, for females, higher adherence to the processed dietary pattern was positively associated with higher levels of Tl and Mo, while being negatively associated with Sn. Greater adherence to the balanced dietary pattern showed a significant positive relationship with Co. Notably, higher adherence to the traditional dietary pattern was negatively associated with V, Mn, Ni, Sn, and Zn. Furthermore, higher adherence to the animal offal dietary pattern was negatively associated with Ni and Se, while being positively associated with Mo. In males, higher adherence to the processed dietary pattern was negatively associated with V, Ni, Sn, and Ag. The balanced dietary pattern showed negative associations with Li, Co, and Ag. Additionally, we also observed that greater adherence to the traditional dietary pattern was negatively associated with Al, Mn, and Ba (Supplementary Tables S5, S6).

## 4 Discussion

In this prospective cohort study, we explored the association between dietary intake and 21 trace elements in couples undergoing IVF treatment. The couple-based analysis revealed significant associations between dietary intake and trace elements after adjusting for shared confounding factors. Moderate whole grain consumption is positively associated with Al, V, Mn, Sn, and Ag. Additionally, dark vegetables and fruits intake was associated with increased Ce and Ga levels, while legumes and soy products showed significant association with Al and Mo. Furthermore, red meat consumption demonstrated a negative association with Tl, yet a positive association with Sn, Ce, and Se. Animal offal and processed meats were also found to be associated with increased levels of Al and Sr. Sugar-sweetened beverages were inversely associated with Tl levels, whereas pickled and fried foods, as well as coffee, were positively associated with Al and Mn. Consumption of tea was associated with increased levels of Co, Ga, and Sr.

In partner-specific models, females showed no association between dietary intake and the concentration of trace elements after FDR correction. However in males, tea and coffee intake was positively correlated with Rb and Ag levels both before and after FDR adjustment. From a translational perspective, although the present findings are observational in nature, they may offer several practical implications for dietary management in couples undergoing IVF. First, while whole grains, dark vegetables, and legumes are nutritionally beneficial and indispensable for reproductive health, their positive associations with certain trace metals (e.g., Al, Mo, and V) suggest that greater diversification of food sources and varieties may help avoid cumulative exposure from specific agricultural or environmental inputs. Second, given that tea and coffee consumption in males remained significantly associated with higher levels of Rb and Ag even after stringent multiple-testing correction, moderating the intake of these beverages during the preconception period may be a prudent consideration to protect the seminal microenvironment. Third, to reduce exposure to so-called “hidden” metals such as Sn that are often related to food contact materials and processing, prioritizing fresh and minimally processed foods over canned or highly processed products may represent a reasonable precautionary strategy. Finally, in light of the strong within-couple concordance observed for both dietary patterns and internal metal profiles, these dietary adjustments may be more effective when implemented at the household level rather than by one partner alone. Collectively, these considerations should be interpreted as supportive, exposure-conscious dietary guidance rather than prescriptive clinical recommendations, and warrant confirmation in future interventional and outcome-oriented studies. The observed sex-specific associations between dietary patterns and internal metal profiles in the present study are biologically plausible and may reflect well-recognized sex differences in metal absorption, distribution, metabolism, and susceptibility. A well-established mechanism for sex differences in internal dose is iron-status–dependent intestinal metal transport, because iron deficiency upregulates divalent metal transport pathways (e.g., DMT1-related uptake), which can increase absorption and retention of certain metals and is more common in women of reproductive age (26). Beyond toxicokinetics, sex hormones can directly modulate metal-related molecular actions. For example, cadmium has been shown to activate estrogen receptor signaling *in vitro*, supporting the broader concept that the hormonal milieu can modify metal bioactivity and downstream cellular responses (27). Consistently, *in vivo* evidence indicates that cadmium can mimic estrogenic effects in hormone-responsive tissues, illustrating how endocrine context may shape tissue distribution and biological responses even at comparable exposure levels (28). Oxidative-stress biology represents another plausible axis underlying sex disparity. Experimental studies have shown that female mitochondria exhibit higher expression of antioxidant genes and lower oxidative damage than those of males, which may alter redox-sensitive handling and toxicity of metals acting through reactive oxygen species–related mechanisms (29). In addition, sex-specific variation in antioxidant enzyme activity has been reported across organs and brain regions in animal models, further supporting inherent sex differences in baseline redox defense capacity (30). Estrogen-associated neuroprotective and antioxidant effects have also been documented in neuronal models, reinforcing the plausibility that hormonal signaling can influence oxidative-injury thresholds relevant to metal toxicology (31). Regarding arsenic as an illustrative example of sex-modified outcomes and mechanisms, experimental evidence shows marked sex differences in tumor spectra following prenatal inorganic arsenic exposure, highlighting that sex can shape long-term susceptibility and target-organ specificity (32). In this context, the sex-specific associations observed in our diet–biomarker analyses (e.g., Mo-related patterns in females and Co-related patterns in males) appear biologically plausible and may reflect sex differences in gastrointestinal absorption and transport, hormone-regulated distribution, and redox regulatory capacity. Taken together, although our study is observational and cannot establish causality, converging toxicological and epidemiological evidence supports the notion that sex-related differences in metal metabolism and susceptibility may, at least in part, underlie the sex-specific associations observed in our results.

Diet has been widely acknowledged as an important source of trace elements. Studies indicate that cereals are the primary contributors to the intake of Al, V, and Mn (33–35). Furthermore, Ce, a common rare earth element, is typically found in higher concentrations in foods (36), which aligns with the findings of this study. However, existing research has yet to clearly identify the main dietary sources of Sn and Ga. A Japanese study has noted that the primary dietary sources of Mo include rice, soybean products, and plant-based foods (37). Meat is recognized as a significant source of Se (38), and both meat and seafood have been reported to contain high levels of Sn and Ce (38, 39). Consistent with the results of our study, traditional Chinese food, such as youtiao (fried dough sticks), is considered one of the dietary sources of Al (40). Notably, several studies have demonstrated that tea is rich in Co and Sr. (40–42).

Moreover, the analysis revealed various associations between dietary patterns and trace elements in both couple-based and partner-specific models. In the couple-based model, higher adherence to the beverage pattern was positively associated with Al, Ga, and Ba, while the processed pattern was associated with Ni. In the partner-specific model, females showed a positive association between higher adherence to the processed pattern and Tl and Mo, whereas males had negative associations with V, Ni, and Ag in the processed pattern. Greater adherence to the traditional dietary pattern consistently showed negative associations with several trace elements across both models, such as Al, Mn, and Ba.

Dietary patterns provide a comprehensive reflection of an individual’s daily eating habits, particularly considering the diversity of the diet. To our knowledge, research on the relationship between dietary patterns and trace metal elements is relatively limited, with most studies focusing on common metals such as Pb, Cd, and Hg (4, 43–45). Due to the diversity of foods within dietary patterns, the positive and negative relationships between dietary patterns and trace elements may potentially offset each other. For instance, in processed dietary patterns, we found a negative association with Sn in both females and males. However, this relationship was not observed in analyses based on couples. Furthermore, analyses of individual food types indicated a positive association between Sn and pickled, fried foods, and sugary beverages among females. This phenomenon may be attributed to the offsetting negative influences of other food types within the processed dietary pattern on Sn levels. Gender differences may be significant factors influencing food choices and dietary patterns, leading to variations in trace element intake. For example, there are notable differences in food preferences and choices between men and women, which can affect the overall formation of dietary patterns. Moreover, multiple studies have confirmed a significant association between trace metal elements and IVF outcomes (9, 24, 46, 47). Importantly, diet may play a critical role in this relationship. By providing guidance and interventions regarding the dietary choices of both partners undergoing IVF, it is possible to effectively help them reduce the potential impact of trace metal elements on fertility, thereby optimizing treatment outcomes. Notably, some of the observed associations between specific food groups and trace elements (e.g., legumes-Al, coffee-Mn) are not readily interpretable from a classical nutritional or toxicological perspective. These findings should not be understood as direct causal effects of these foods per se, but more likely reflect the complexity of food matrices, contamination during cultivation, processing or storage, or shared environmental exposure sources (e.g., soil, water, and packaging materials). For less frequently discussed elements such as Ce, Ga, Ag, and Ba, we did not intend to overinterpret their biological relevance. These elements were included because they are environmentally relevant but understudied components of the human exposome and can enter the body through dietary and environmental pathways. Their analysis reflects the comprehensive scope of the exposure assessment rather than *a priori* hypotheses regarding reproductive toxicity.

Our study has several strengths. First, we conducted a multicenter prospective cohort study to measure the concentrations of 21 trace metal elements in couples, thereby providing a more comprehensive exploration of the impact of diet on the levels of these trace metals. Second, we studied 13 food types, which cover almost the main components of the daily diet of Chinese people. In addition, factor analysis was further used to explore the effects of different dietary patterns on the levels of trace metals. Finally, based on the large sample size study design, we can more accurately assess the dietary influence of couples on plasma trace metal concentrations, thus providing a scientific basis for optimizing nutritional interventions related to fertility.

However, our study has several limitations. First, we estimated the joint exposure levels of partners by calculating the average concentrations of trace elements in both partners, a method that may not be entirely accurate. Although this approach has been utilized in previous studies (25), further research is needed to evaluate more precise methods for assessing couples’ exposure. Second, the dietary intake data in our study were obtained through self-reported questionnaires, which may introduce potential recall bias. Additionally, our dietary assessment was based on a simplified 14-item FFQ, which captures only the frequency of intake without accounting for specific portion sizes. This simplified tool may lead to some degree of measurement error or misclassification in absolute nutrient intake. However, this approach was intentionally adopted to minimize respondent burden and ensure high completion rates and feasibility across our large, multicenter cohort. While it lacks the granularity of more comprehensive dietary records, it remains a robust method for identifying and categorizing major dietary patterns, which was the primary objective of this study. To enhance the accuracy of future research, further optimization of the questionnaire design is needed to more accurately estimate the impact of diet on trace element exposure. Third, from a clinical translation perspective, it is important to emphasize that the primary objective of the present study was to characterize how specific dietary habits and overall dietary patterns relate to internal trace element profiles in couples undergoing IVF, rather than to evaluate the downstream associations of these elements with reproductive outcomes. Accordingly, the current findings should be viewed as reflecting relatively “upstream” exposure determinants and are mainly hypothesis-generating in nature. We did not formally model trace element concentrations as predictors of key IVF outcomes such as embryo quality, implantation, or live birth, which limits the direct clinical interpretability of our results for patient counseling at this stage. Nevertheless, by identifying potentially modifiable dietary determinants of trace element status in the IVF setting, our results provide an important foundation for subsequent stepwise investigations, first linking diet to biomarkers and then linking these biomarkers to clinically meaningful reproductive endpoints. Future analyses within this cohort will formally evaluate whether the diet-related trace elements identified here are associated with key IVF outcomes, thereby helping to establish a more complete and clinically actionable evidence chain.

Finally, our study population consists of couples undergoing IVF treatment at the reproductive centers, which limits the generalizability of our results to the general population. Nevertheless, this research provides valuable insights for couples facing fertility issues, helping them understand the potential role of dietary factors in trace metal element exposure and offering reference points for future nutritional interventions.

## 5 Conclusion

In the couple-based study, we revealed significant associations between dietary intake and 21 trace metal elements in couples undergoing IVF treatment, highlighting the complex interplay between nutrition and metal exposure. Specifically, moderate or high consumption of coarse grains, dark-colored vegetables, fruits, and legumes was associated with increased levels of various trace metals, such as Al, V, Mn, Sn, Ag, Ce, and Mo, while red meat and processed foods exhibited distinct relationships with different metals by gender. Notably, various dietary patterns, including processed, traditional, balanced, beverage, and single animal offal dietary patterns, showed different associations with multiple trace metal elements in both couple and partner-specific models. These findings highlight the importance of dietary factors in understanding trace metal exposure, provide valuable insights for couples facing fertility challenges, and inform future nutritional interventions aimed at reducing the risk of exposure. Further studies are needed to explore these associations in a broader population and to refine dietary assessments.

## Data Availability

The raw data supporting the conclusions of this article will be made available by the authors, without undue reservation.
