# Supplementary material for: Association between diet intake and trace elements concentrations in couples undergoing in vitro fertilization: a couple-based exploration
Source: Front Nutr. 2026 Feb 12;13:1722802. doi: 10.3389/fnut.2026.1722802 (PMC12935679; doi:10.3389/fnut.2026.1722802)
Supplement: Supplementary file 1 [file Data_Sheet_1.PDF]

Association between Diet Intake and Trace Elements Concentrations in Couples Undergoing in vitro fertilization: A Couple-Based Exploration

Table of Contents

Table S1. Detection and distribution of plasma trace elements concentrations in couples (N=1,066).

Table S2. Factor loading matrix of the four identified dietary patterns for females.

Table S3. Factor loading matrix of the three identified dietary patterns for males.

Table S4. Factor loading matrix of the four identified dietary patterns (Couple-based).

Table S5. Association between dietary patterns and trace elements concentrations among 1,066 females.

Table S6. Association between dietary patterns and trace elements concentrations among 1,066 males.

Table S7. Association between dietary intake and trace elements concentrations among 1,066 couples.

Table S8. Association between dietary intake and trace elements concentrations among 1,066 females.

Table S9. Association between dietary intake and trace elements concentrations among 1,066 males.

Figure of Contents

Figure S1. The flowchart of study participants included in the present study.

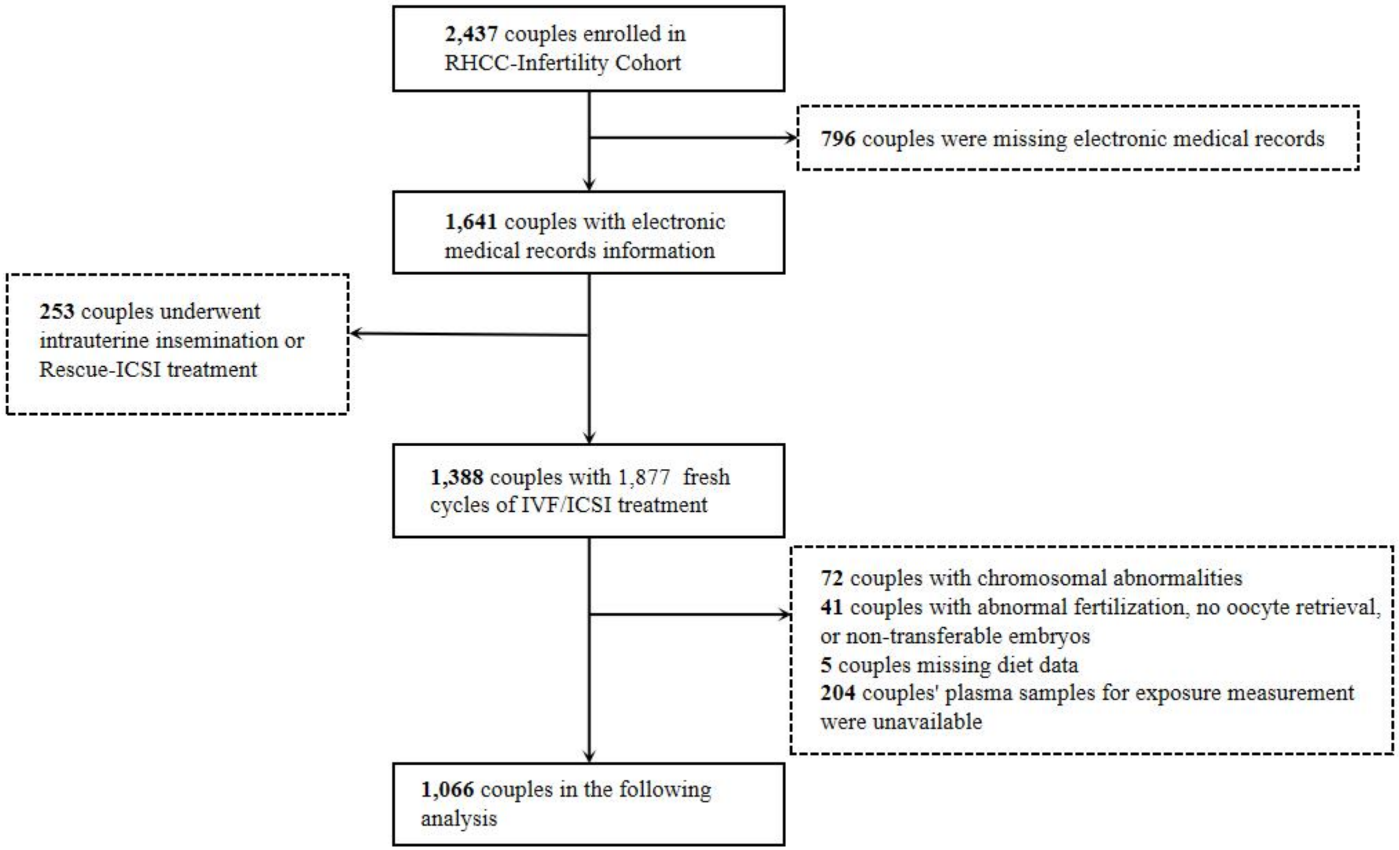

Figure S1. The flowchart of study participants included in the present study.

Table S1. Detection and distribution of plasma trace elements concentrations in couples (N=1,066).

Element	LOD (ng/mL)	N (%) > LOD	Median (Q1, Q3)
**Male**			
Be	0.04702	580 (54.4)	0.13(0.03,0.21)
Al	0.68935	1,066(100.0)	67.73(47.81,91.47)
V	0.00003	1,066(100.0)	0.28(0.20,0.40)
Li	0.02264	1,066(100.0)	3.49(2.08,6.84)
Fe	0.74444	1,066(100.0)	4338.89(3517.05,5252.58)
Mn	0.06224	1,066(100.0)	1.05(0.86,1.34)
Co	0.00230	1,066(100.0)	0.15(0.13,0.17)
Ni	0.04836	1,066(100.0)	1.38(1.10,1.93)
Ga	0.00469	1,066(100.0)	0.52(0.40,0.68)
Rb	0.00191	1,066(100.0)	228.29(200.87,256.46)

Sn	0.66401	1,058 (99.2)	4.02(2.63,6.27)
Sr	0.12808	1,066(100.0)	36.66(31.04,43.79)
Ce	0.07242	1,066(100.0)	150.27(123.00,196.01)
Cu	0.31070	1,066(100.0)	3.27(2.89,3.696)
Ag	0.00429	1,066(100.0)	0.11(0.08,0.17)
Cd	0.00163	1,065(99.9)	0.05(0.03,0.10)
Ba	0.62839	1,066(100.0)	64.05(50.32,76.98)
Se	0.02477	1,066(100.0)	93.61(83.15,104.78)
Zn	3.46262	1,066(100.0)	4207.52(3299.93,6346.42)
Tl	0.00032	1,066(100.0)	0.13(0.11,0.18)
Mo	0.43904	1,066(100.0)	26.57(21.17,33.17)
**Female**			
Be	0.04702	562 (52.7)	0.13(0.03,0.21)

Al	0.68935	1,066(100.0)	61.92(43.73,89.87)
V	0.00003	1,066(100.0)	0.25(0.18,0.37)
Li	0.02264	1,058(99.2)	3.04(1.84,5.85)
Fe	0.74444	1,066(100.0)	3381.95(2593.16,4258.70)
Mn	0.06224	1,066(100.0)	0.98(0.77,1.29)
Co	0.00230	1,066(100.0)	0.22(0.16,0.33)
Ni	0.04836	1,066(100.0)	1.36(1.06,1.85)
Ga	0.00469	1,066(100.0)	0.48(0.37,0.62)
Rb	0.00191	1,066(100.0)	200.41(176.82,224.11)
Sn	0.66401	1,057(99.2)	3.72(2.50,5.73)
Sr	0.12808	1,066(100.0)	36.20(30.17,43.77)
Ce	0.07242	1,066(100.0)	137.86(113.98,177.15)
Cu	0.31070	1,066(100.0)	3.77(3.29,4.33)

Ag	0.00429	1,066(100.0)	0.11(0.08,0.17)
Cd	0.00163	1,064(99.8)	0.05(0.03,0.08)
Ba	0.62839	1,066(100.0)	57.88(46.23,71.57)
Se	0.02477	1,066(100.0)	89.01(78.78,100.34)
Zn	3.46262	1,066(100.0)	3933.25(3102.18,5753.65)
Tl	0.00032	1,066(100.0)	0.13(0.10,0.17)
Mo	0.43904	1,066(100.0)	25.42(21.08,30.22)

Notes: Only trace metal elements detected in >50 of each partner were included in the final association analyses. LOD: limits of detection; Q1 and Q3 refer to the 1st and 3rd quartile.

Table S2 Factor loading matrix of the four identified dietary patterns for females.

Food Items	Factor 1 (Mixed/SSB)	Factor 2 (Prudent)	Factor 3 (Traditional)	Factor 4 (Niche)
White rice	-0.02	0.02	0.46	0.02
Whole grains	0.26	0.6	-0.02	0.1
Dark vegetables	0.01	0.33	0.46	-0.05
Fruits	0.02	0.42	0.32	-0.1
Legumes and legume products	0.07	0.61	0.29	0.08
Nuts	0.32	0.58	0.13	0.21
Red meat	0.14	0.13	0.5	0.12
Animal viscera	0.51	0.28	0.17	0.52
Processed meat	0.68	0.16	0.09	0.3
Sugar-sweetened beverages	0.78	0.04	0.07	-0.03
Pickled or fried foods	0.68	0.02	0.19	0.02
Coffee	0.66	0.23	-0.08	0.1
Tea	0.45	0.19	-0.06	0.05

Table S3 Factor loading matrix of the three identified dietary patterns for males.

Food Items	Factor 1 (Processed Pattern)	Factor 2 (Prudent Pattern)	Factor 3 (Traditional Pattern)
White rice	-0.02	0.04	0.46
Whole grains	0.27	0.63	0.03
Dark vegetables	0.01	0.25	0.48
Fruits	0.04	0.55	0.33
Legumes and legume products	0.17	0.45	0.39
Nuts	0.32	0.68	0.15
Red meat	0.16	0.05	0.52
Animal viscera	0.61	0.31	0.1
Processed meat	0.68	0.32	0.03
Sugar-sweetened beverages	0.67	0.03	0.05
Pickled or fried foods	0.73	0.06	0.22
Coffee	0.64	0.33	-0.02
Tea	0.2	0.17	0.2

Table S4 Factor loading matrix of the four identified dietary patterns (Couple-based)

Food Items	Factor 1((Beverage)	Factor 2 (Processed)	Factor 3 (Prudent)	Factor 4 (Traditional)
White rice	0.04	0.01	0.02	0.45
Whole grains	0.16	0.31	0.53	-0.02
Dark vegetables	0.05	0.01	0.35	0.44
Fruits	0.05	0.04	0.45	0.19
Legumes and legume products	0.05	0.19	0.53	0.2
Nuts	0.11	0.45	0.55	0.05
Red meat	-0.02	0.14	0.13	0.51
Animal viscera	0.16	0.64	0.35	0.05
Processed meat	0.17	0.75	0.26	0.03
Sugar-sweetened beverages	0.21	0.66	0.08	0.08
Pickled or fried foods	0.11	0.73	0.12	0.14
Coffee	0.82	0.54	0.17	0.00
Tea	0.28	0.27	0.17	0.11

Table S5 Association between dietary patterns and trace elements concentrations among 1,066 females.

Element	Processed dietary pattern			Balanced dietary pattern			Traditional dietary pattern			Animal offal dietary pattern		
	T1	T2	T3	T1	T2	T3	T1	T2	T3	T1	T2	T3
Be	Ref	-0.06(-0.21,0.08)	0.03(-0.12,0.18)	Ref	0.06(-0.09,0.21)	0.13(-0.02,0.29)	Ref	0.02(-0.13,0.17)	-0.05(-0.21,0.10)	Ref	0.03(-0.12,0.18)	-0.13(-0.27,0.02)
Al	Ref	0.03(-0.12,0.18)	-0.03(-0.19,0.12)	Ref	-0.06(-0.20,0.09)	-0.04(-0.20,0.11)	Ref	-0.08(-0.23,0.06)	-0.03(-0.18,0.12)	Ref	0.00(-0.14,0.15)	-0.12(-0.26,0.03)
V	Ref	-0.03(-0.17,0.11)	0.03(-0.18,0.11)	Ref	-0.08(-0.22,0.07)	0.09(-0.06,0.23)	Ref	**-0.14(-0.28,-0.00)**	-0.01(-0.16,0.14)	Ref	-0.05(-0.19,0.10)	-0.03(-0.17,0.11)
Li	Ref	-0.00(-0.15,0.15)	-0.05(-0.20,0.09)	Ref	-0.07(-0.22,0.07)	0.02(-0.13,0.17)	Ref	-0.12(-0.26,0.03)	0.05(-0.10,0.20)	Ref	-0.09(-0.24,0.05)	-0.05(-0.20,0.09)
Fe	Ref	-0.03(-0.18,0.12)	0.02(-0.13,0.17)	Ref	-0.12(-0.26,0.03)	-0.05(-0.20,0.10)	Ref	-0.00(-0.15,0.14)	-0.06(-0.21,0.09)	Ref	0.05(-0.10,0.20)	0.03(-0.12,0.18)
Mn	Ref	-0.10(-0.24,0.05)	-0.02(-0.17,0.13)	Ref	-0.06(-0.21,0.08)	0.04(-0.11,0.19)	Ref	**-0.17(-0.31,-0.02)**	-0.07(-0.22,0.08)	Ref	-0.08(-0.22,0.07)	0.01(-0.13,0.16)
Co	Ref	-0.06(-0.21,0.08)	-0.07(-0.22,0.08)	Ref	**0.18(0.04,0.32)**	0.02(-0.13,0.17)	Ref	-0.03(-0.17,0.12)	0.04(-0.11,0.18)	Ref	-0.04(-0.18,0.10)	0.00(-0.14,0.15)
Ni	Ref	-0.03(-0.18,0.11)	-0.07(-0.22,0.08)	Ref	0.04(-0.11,0.19)	0.07(-0.09,0.22)	Ref	**-0.16(-0.30,-0.01)**	-0.10(-0.25,0.05)	Ref	**-0.21(-0.36,-0.07)**	-0.10(-0.25,0.04)
Ga	Ref	-0.00(-0.15,0.14)	0.03(-0.12,0.17)	Ref	0.02(-0.12,0.16)	-0.04(-0.19,0.10)	Ref	-0.06(-0.21,0.08)	-0.09(-0.24,0.06)	Ref	0.13(-0.01,0.27)	0.03(-0.11,0.17)

Rb	Ref	-0.07(-0.22,0.07)	-0.12(-0.26,0.03)	Ref	0.03(-0.12,0.17)	-0.00(-0.15,0.15)	Ref	-0.03(-0.17,0.11)	-0.10(-0.25,0.04)	Ref	0.05(-0.09,0.19)	-0.07(-0.21,0.07)
Sn	Ref	-0.13(-0.28,0.01)	**-0.15(-0.30,-0.00)**	Ref	-0.08(-0.23,0.07)	-0.06(-0.22,0.09)	Ref	-0.08(-0.22,0.07)	**-0.19(-0.34-0.04)**	Ref	0.14(-0.00,0.29)	0.06(-0.08,0.21)
Sr	Ref	-0.05(-0.19,0.10)	0.01(-0.13,0.16)	Ref	-0.01(-0.16,0.13)	0.03(-0.12,0.18)	Ref	-0.06(-0.20,0.09)	0.04(-0.11,0.18)	Ref	-0.12(-0.26,0.03)	-0.07(-0.21,0.08)
Ce	Ref	-0.00(-0.15,0.14)	0.03(-0.11,0.18)	Ref	-0.04(-0.18,0.11)	-0.11(-0.26,0.04)	Ref	-0.09(-0.23,0.06)	-0.10(-0.25,0.05)	Ref	0.12(-0.02,0.27)	0.09(-0.06,0.23)
Cu	Ref	0.02(-0.12,0.16)	0.09(-0.06,0.23)	Ref	0.00(-0.14,0.14)	0.06(-0.08,0.21)	Ref	0.03(-0.12,0.16)	-0.00(-0.15,0.14)	Ref	0.01(-0.13,0.15)	-0.03(-0.17,0.11)
Ag	Ref	-0.05(-0.19,0.10)	-0.09(-0.23,0.06)	Ref	-0.02(-0.17,0.13)	0.14(-0.01,0.29)	Ref	-0.00(-0.15,0.15)	0.03(-0.12,0.18)	Ref	-0.02(-0.16,0.13)	0.02(-0.13,0.17)
Cd	Ref	0.06(-0.09,0.21)	0.15(-0.00,0.30)	Ref	0.06(-0.09,0.21)	0.00(-0.15,0.15)	Ref	-0.16(-0.31,-0.02)	-0.04(-0.19,0.12)	Ref	-0.12(-0.27,0.02)	0.00(-0.14,0.15)
Ba	Ref	0.05(-0.10,0.19)	-0.07(-0.22,0.07)	Ref	0.00(-0.14,0.15)	-0.03(-0.19,0.12)	Ref	-0.13(-0.28,0.02)	-0.13(-0.28,0.02)	Ref	-0.04(-0.18,0.11)	-0.06(-0.21,0.09)
Se	Ref	-0.09(-0.24,0.05)	-0.02(-0.16,0.13)	Ref	0.03(-0.12,0.17)	0.09(-0.06,0.24)	Ref	0.01(-0.14,0.15)	-0.04(-0.18,0.11)	Ref	0.02(-0.12,0.17)	**-0.15(-0.30,-0.01)**
Zn	Ref	-0.04(-0.19,0.10)	-0.04(-0.19,0.11)	Ref	-0.09(-0.23,0.06)	0.03(-0.12,0.17)	Ref	**-0.18(-0.33,-0.04)**	-0.07(-0.22,0.08)	Ref	-0.07(-0.21,0.08)	-0.02(-0.16,0.13)
Tl	Ref	**0.19(0.05,0.34)**	0.07(-0.08,0.22)	Ref	0.06(-0.08,0.21)	0.02(-0.13,0.17)	Ref	0.04(-0.11,0.19)	-0.03(-0.18,0.12)	Ref	0.06(-0.09,0.20)	0.06(-0.09,0.21)
Mo	Ref	0.02(-0.03,0.06)	**0.06(0.02,0.11)**	Ref	0.00(-0.04,0.05)	0.01(-0.04,0.05)	Ref	-0.04(-0.08,0.01)	-0.03(-0.08,0.01)	Ref	0.02(-0.02,0.07)	**0.05(0.00,0.09)**

Note: Ref, Reference; To enhance readability and clarity, we have simplified the tables to highlight only the positive results.

Table S6 Association between dietary patterns and trace elements concentrations among 1,066 males.

Element	Processed dietary pattern			Balanced dietary pattern			Traditional dietary pattern		
	T1	T2	T3	T1	T2	T3	T1	T2	T3
Be	Ref	0.00(-0.15,0.15)	-0.02(-0.17,0.13)	Ref	0.00(-0.14,0.15)	0.07(-0.08,0.23)	Ref	0.09(-0.06,0.24)	0.04(-0.11,0.19)
Al	Ref	-0.14(-0.29,0.01)	-0.15(-0.30,0.00)	Ref	-0.07(-0.21,0.08)	-0.00(-0.16,0.15)	Ref	**-0.15(-0.30,-0.00)**	-0.12(-0.27,0.03)
V	Ref	-0.06(-0.20,0.08)	**-0.16(-0.30,-0.02)**	Ref	-0.08(-0.21,0.06)	0.06(-0.09,0.20)	Ref	-0.11(-0.25,0.03)	-0.09(-0.23,0.05)
Li	Ref	-0.07(-0.21,0.08)	-0.08(-0.22,0.07)	Ref	**-0.18(-0.33,-0.04)**	-0.03(-0.17,0.12)	Ref	-0.00(-0.15,0.14)	-0.01(-0.15,0.14)
Fe	Ref	-0.12(-0.26,0.03)	-0.06(-0.21,0.10)	Ref	-0.01(-0.16,0.14)	0.13(-0.02,0.28)	Ref	-0.02(-0.16,0.13)	-0.03(-0.18,0.12)
Mn	Ref	-0.02(-0.16,0.13)	-0.08(-0.23,0.07)	Ref	-0.08(-0.22,0.07)	0.07(-0.07,0.22)	Ref	-0.08(-0.22,0.06)	**-0.15(-0.30,-0.01)**
Co	Ref	-0.09(-0.24,0.06)	-0.07(-0.22,0.08)	Ref	**-0.19(-0.34,-0.04)**	**-0.22(-0.37,-0.07)**	Ref	-0.08(-0.23,0.06)	-0.10(-0.25,0.05)
Ni	Ref	**-0.16(-0.31,-0.02)**	-0.02(-0.17,0.13)	Ref	-0.06(-0.21,0.09)	-0.05(-0.20,0.10)	Ref	0.04(-0.10,0.19)	0.02(-0.13,0.17)
Ga	Ref	-0.00(-0.15,0.14)	0.04(-0.10,0.19)	Ref	0.01(-0.13,0.16)	-0.05(-0.20,0.09)	Ref	-0.10(-0.25,0.04)	-0.08(-0.23,0.06)
Rb	Ref	0.04(-0.11,0.18)	0.10(-0.05,0.25)	Ref	-0.04(-0.18,0.11)	0.07(-0.08,0.22)	Ref	-0.05(-0.19,0.10)	-0.13(-0.27,0.02)

Sn	Ref	-0.10(-0.25,0.04)	**-0.15(-0.30,-0.00)**	Ref	-0.04(-0.19,0.10)	-0.07(-0.22,0.07)	Ref	-0.06(-0.21,0.08)	-0.09(-0.24,0.06)
Sr	Ref	-0.07(-0.22,0.07)	-0.06(-0.21,0.08)	Ref	-0.05(-0.19,0.09)	0.03(-0.11,0.08)	Ref	-0.12(-0.26,0.02)	-0.14(-0.28,0.01)
Ce	Ref	-0.03(-0.17,0.12)	0.04(-0.11,0.19)	Ref	0.01(-0.14,0.15)	-0.04(-0.18,0.11)	Ref	-0.12(-0.27,0.02)	-0.07(-0.22,0.07)
Cu	Ref	0.10(-0.04,0.24)	-0.08(-0.22,0.07)	Ref	0.01(-0.13,0.15)	0.09(-0.05,0.24)	Ref	-0.00(-0.14,0.13)	-0.04(-0.18,0.10)
Ag	Ref	**-0.19(-0.34-0.05)**	**-0.21(-0.36,-0.06)**	Ref	-0.09(-0.24,0.05)	**-0.15(-0.30,-0.00)**	Ref	-0.09(-0.24,0.05)	0.00(-0.14,0.15)
Cd	Ref	-0.00(-0.15,0.14)	-0.02(-0.16,0.13)	Ref	0.01(-0.13,0.15)	0.02(-0.12,0.17)	Ref	-0.07(-0.22,0.07)	-0.06(-0.21,0.08)
Ba	Ref	-0.09(-0.24,0.06)	-0.05(-0.20,0.10)	Ref	-0.02(-0.17,0.13)	0.01(-0.14,0.16)	Ref	**-0.16(-0.30,-0.01)**	-0.04(-0.19,0.11)
Se	Ref	-0.03(-0.17,0.12)	0.01(-0.14,0.16)	Ref	-0.18(-0.32,-0.03)	-0.07(-0.22,0.08)	Ref	0.01(-0.14,0.15)	-0.11(-0.25,0.04)
Zn	Ref	-0.03(-0.18,0.12)	-0.10(-0.24,0.05)	Ref	-0.10(-0.25,0.04)	-0.02(-0.17,0.13)	Ref	-0.13(-0.27,0.02)	-0.12(-0.27,0.02)
Tl	Ref	-0.05(-0.20,0.09)	-0.07(-0.22,0.08)	Ref	-0.03(-0.18,0.12)	0.07(-0.08,0.22)	Ref	0.09(-0.06,0.23)	0.12(-0.03,0.26)
Mo	Ref	-0.02(-0.07,0.03)	0.04(-0.02,0.09)	Ref	0.03(-0.02,0.08)	0.04(-0.02,0.09)	Ref	0.01(-0.04,0.06)	-0.02(-0.08,0.03)

Note: Ref, Reference; To enhance readability and clarity, we have simplified the tables to highlight only the positive results.

Table S7 Association between dietary intake and trace elements concentrations among 1,066 couples.

Trace elements-Couple	Dietary	Level	Beta (95% CI)	P-value	P-FDR
Be.C	White rice	moderate	-0.10 (-0.36, 0.17)	0.48	0.95
Be.C	White rice	high	-0.08 (-0.34, 0.18)	0.53	0.95
Be.C	Coarse grain	moderate	0.04 (-0.10, 0.18)	0.57	0.95
Be.C	Coarse grain	high	0.09 (-0.17, 0.35)	0.50	0.95
Be.C	Dark-Colored vegetables	moderate	-0.03 (-0.22, 0.16)	0.76	0.97
Be.C	Dark-Colored vegetables	high	-0.06 (-0.24, 0.13)	0.54	0.95
Be.C	Fruits	moderate	0.00 (-0.16, 0.16)	0.99	0.99
Be.C	Fruits	high	-0.06 (-0.23, 0.11)	0.50	0.95
Be.C	Legumes and soy products	moderate	0.12 (-0.01, 0.26)	0.07	0.95
Be.C	Legumes and soy products	high	0.01 (-0.16, 0.19)	0.88	0.99
Be.C	Nuts	moderate	-0.11 (-0.25, 0.03)	0.14	0.95
Be.C	Nuts	high	-0.08 (-0.32, 0.16)	0.52	0.95
Be.C	Red meat	moderate	0.08 (-0.11, 0.27)	0.41	0.95
Be.C	Red meat	high	-0.03 (-0.22, 0.16)	0.76	0.97
Be.C	Animal offal	moderate	0.03 (-0.13, 0.18)	0.75	0.97
Be.C	Animal offal	high	-0.00 (-0.34, 0.33)	0.99	0.99
Be.C	Processed meat	moderate	0.04 (-0.12, 0.19)	0.63	0.97
Be.C	Processed meat	high	-0.25 (-0.62, 0.12)	0.18	0.95
Be.C	Sugar-sweetened beverages	moderate	-0.11 (-0.25, 0.03)	0.13	0.95
Be.C	Sugar-sweetened beverages	high	-0.13 (-0.41, 0.15)	0.35	0.95
Be.C	Pickled or fried food	moderate	-0.01 (-0.16, 0.13)	0.84	0.99
Be.C	Pickled or fried food	high	-0.08 (-0.35, 0.20)	0.58	0.95

Be.C	Coffee	moderate	0.08 (-0.09, 0.26)	0.36	0.95
Be.C	Coffee	high	-0.14 (-0.58, 0.30)	0.52	0.95
Be.C	Tea	moderate	-0.02 (-0.16, 0.11)	0.73	0.97
Be.C	Tea	high	0.01 (-0.20, 0.22)	0.93	0.99
Al.C	White rice	moderate	-0.06 (-0.32, 0.20)	0.65	0.70
Al.C	White rice	high	-0.00 (-0.26, 0.25)	0.99	0.99
Al.C	Coarse grain	moderate	0.16 (0.02, 0.29)	0.02	0.13
Al.C	Coarse grain	high	0.08 (-0.17, 0.34)	0.52	0.70
Al.C	Dark-Colored vegetables	moderate	0.05 (-0.13, 0.24)	0.58	0.70
Al.C	Dark-Colored vegetables	high	0.12 (-0.06, 0.30)	0.20	0.46
Al.C	Fruits	moderate	-0.06 (-0.22, 0.10)	0.44	0.66
Al.C	Fruits	high	0.07 (-0.10, 0.23)	0.44	0.66
Al.C	Legumes and soy products	moderate	0.04 (-0.10, 0.17)	0.59	0.70
Al.C	Legumes and soy products	high	0.17 (0.00, 0.34)	0.05	0.20
Al.C	Nuts	moderate	0.06 (-0.08, 0.20)	0.40	0.66
Al.C	Nuts	high	0.06 (-0.18, 0.30)	0.63	0.70
Al.C	Red meat	moderate	0.10 (-0.09, 0.29)	0.30	0.64
Al.C	Red meat	high	0.16 (-0.03, 0.34)	0.10	0.32
Al.C	Animal offal	moderate	0.23 (0.07, 0.38)	0.00	0.04
Al.C	Animal offal	high	0.14 (-0.19, 0.47)	0.42	0.66
Al.C	Processed meat	moderate	0.20 (0.04, 0.35)	0.01	0.08
Al.C	Processed meat	high	0.17 (-0.20, 0.53)	0.37	0.66
Al.C	Sugar-sweetened beverages	moderate	-0.05 (-0.19, 0.09)	0.45	0.66
Al.C	Sugar-sweetened beverages	high	0.23 (-0.04, 0.51)	0.10	0.32
Al.C	Pickled or fried food	moderate	0.23 (0.09, 0.37)	0.00	0.03
Al.C	Pickled or fried food	high	-0.02 (-0.29, 0.25)	0.91	0.94

Al.C	Coffee	moderate	0.27 (0.10, 0.45)	0.00	0.03
Al.C	Coffee	high	0.47 (0.04, 0.91)	0.03	0.16
Al.C	Tea	moderate	0.03 (-0.10, 0.17)	0.61	0.70
Al.C	Tea	high	0.14 (-0.07, 0.35)	0.19	0.46
V.C	White rice	moderate	0.07 (-0.17, 0.32)	0.55	0.81
V.C	White rice	high	0.07 (-0.18, 0.31)	0.60	0.81
V.C	Coarse grain	moderate	0.14 (0.01, 0.27)	0.03	0.65
V.C	Coarse grain	high	0.11 (-0.13, 0.36)	0.36	0.81
V.C	Dark-Colored vegetables	moderate	0.00 (-0.17, 0.18)	0.97	0.97
V.C	Dark-Colored vegetables	high	0.00 (-0.17, 0.18)	0.97	0.97
V.C	Fruits	moderate	-0.08 (-0.23, 0.07)	0.28	0.81
V.C	Fruits	high	-0.02 (-0.18, 0.14)	0.78	0.91
V.C	Legumes and soy products	moderate	0.04 (-0.09, 0.17)	0.55	0.81
V.C	Legumes and soy products	high	-0.02 (-0.18, 0.15)	0.85	0.91
V.C	Nuts	moderate	-0.05 (-0.18, 0.08)	0.49	0.81
V.C	Nuts	high	0.03 (-0.20, 0.26)	0.82	0.91
V.C	Red meat	moderate	-0.04 (-0.22, 0.14)	0.67	0.81
V.C	Red meat	high	-0.05 (-0.22, 0.13)	0.60	0.81
V.C	Animal offal	moderate	0.09 (-0.06, 0.24)	0.23	0.81
V.C	Animal offal	high	0.14 (-0.17, 0.45)	0.39	0.81
V.C	Processed meat	moderate	0.14 (-0.00, 0.28)	0.05	0.65
V.C	Processed meat	high	-0.27 (-0.61, 0.08)	0.13	0.81
V.C	Sugar-sweetened beverages	moderate	0.06 (-0.07, 0.19)	0.36	0.81
V.C	Sugar-sweetened beverages	high	0.10 (-0.16, 0.36)	0.45	0.81
V.C	Pickled or fried food	moderate	0.12 (-0.01, 0.26)	0.07	0.65
V.C	Pickled or fried food	high	-0.06 (-0.32, 0.20)	0.65	0.81

V.C	Coffee	moderate	0.06 (-0.11, 0.22)	0.50	0.81
V.C	Coffee	high	0.13 (-0.29, 0.54)	0.55	0.81
V.C	Tea	moderate	0.05 (-0.07, 0.18)	0.41	0.81
V.C	Tea	high	0.07 (-0.13, 0.27)	0.47	0.81
Li.C	White rice	moderate	0.20 (-0.06, 0.45)	0.13	0.64
Li.C	White rice	high	0.24 (-0.01, 0.49)	0.06	0.64
Li.C	Coarse grain	moderate	0.09 (-0.04, 0.22)	0.18	0.64
Li.C	Coarse grain	high	0.14 (-0.12, 0.39)	0.29	0.74
Li.C	Dark-Colored vegetables	moderate	0.01 (-0.17, 0.19)	0.92	0.92
Li.C	Dark-Colored vegetables	high	-0.04 (-0.22, 0.14)	0.69	0.81
Li.C	Fruits	moderate	-0.04 (-0.20, 0.11)	0.57	0.74
Li.C	Fruits	high	0.04 (-0.12, 0.21)	0.61	0.74
Li.C	Legumes and soy products	moderate	0.04 (-0.09, 0.17)	0.55	0.74
Li.C	Legumes and soy products	high	0.07 (-0.09, 0.24)	0.39	0.74
Li.C	Nuts	moderate	-0.04 (-0.18, 0.09)	0.55	0.74
Li.C	Nuts	high	0.17 (-0.07, 0.41)	0.16	0.64
Li.C	Red meat	moderate	0.05 (-0.13, 0.23)	0.59	0.74
Li.C	Red meat	high	0.03 (-0.16, 0.21)	0.78	0.84
Li.C	Animal offal	moderate	0.08 (-0.07, 0.24)	0.28	0.74
Li.C	Animal offal	high	0.20 (-0.12, 0.52)	0.22	0.68
Li.C	Processed meat	moderate	0.14 (-0.01, 0.29)	0.07	0.64
Li.C	Processed meat	high	-0.15 (-0.51, 0.20)	0.40	0.74
Li.C	Sugar-sweetened beverages	moderate	0.04 (-0.10, 0.18)	0.55	0.74
Li.C	Sugar-sweetened beverages	high	0.18 (-0.09, 0.45)	0.18	0.64
Li.C	Pickled or fried food	moderate	0.01 (-0.13, 0.15)	0.92	0.92
Li.C	Pickled or fried food	high	0.13 (-0.14, 0.39)	0.35	0.74

Li.C	Coffee	moderate	0.03 (-0.15, 0.20)	0.77	0.84
Li.C	Coffee	high	0.36 (-0.06, 0.79)	0.09	0.64
Li.C	Tea	moderate	0.04 (-0.08, 0.17)	0.50	0.74
Li.C	Tea	high	0.19 (-0.02, 0.40)	0.07	0.64
Fe.C	White rice	moderate	0.19 (-0.07, 0.45)	0.15	0.86
Fe.C	White rice	high	0.10 (-0.16, 0.35)	0.45	0.90
Fe.C	Coarse grain	moderate	-0.01 (-0.15, 0.12)	0.83	0.98
Fe.C	Coarse grain	high	0.11 (-0.15, 0.37)	0.39	0.86
Fe.C	Dark-Colored vegetables	moderate	0.15 (-0.04, 0.33)	0.12	0.86
Fe.C	Dark-Colored vegetables	high	0.06 (-0.12, 0.25)	0.50	0.91
Fe.C	Fruits	moderate	-0.09 (-0.25, 0.07)	0.27	0.86
Fe.C	Fruits	high	0.01 (-0.16, 0.17)	0.94	0.98
Fe.C	Legumes and soy products	moderate	-0.01 (-0.15, 0.12)	0.87	0.98
Fe.C	Legumes and soy products	high	0.10 (-0.07, 0.28)	0.23	0.86
Fe.C	Nuts	moderate	-0.06 (-0.20, 0.08)	0.40	0.86
Fe.C	Nuts	high	-0.04 (-0.28, 0.20)	0.75	0.98
Fe.C	Red meat	moderate	-0.10 (-0.29, 0.08)	0.28	0.86
Fe.C	Red meat	high	-0.02 (-0.21, 0.16)	0.81	0.98
Fe.C	Animal offal	moderate	0.01 (-0.15, 0.16)	0.92	0.98
Fe.C	Animal offal	high	-0.15 (-0.48, 0.18)	0.38	0.86
Fe.C	Processed meat	moderate	-0.01 (-0.16, 0.14)	0.88	0.98
Fe.C	Processed meat	high	0.12 (-0.25, 0.49)	0.52	0.91
Fe.C	Sugar-sweetened beverages	moderate	-0.08 (-0.22, 0.06)	0.25	0.86
Fe.C	Sugar-sweetened beverages	high	-0.12 (-0.39, 0.16)	0.40	0.86
Fe.C	Pickled or fried food	moderate	0.03 (-0.11, 0.17)	0.65	0.96
Fe.C	Pickled or fried food	high	-0.04 (-0.31, 0.23)	0.77	0.98

Fe.C	Coffee	moderate	0.05 (-0.13, 0.22)	0.58	0.94
Fe.C	Coffee	high	0.12 (-0.32, 0.55)	0.60	0.94
Fe.C	Tea	moderate	0.08 (-0.05, 0.21)	0.22	0.86
Fe.C	Tea	high	0.26 (0.05, 0.47)	0.01	0.39
Mn.C	White rice	moderate	0.15 (-0.11, 0.40)	0.25	0.47
Mn.C	White rice	high	0.18 (-0.07, 0.43)	0.15	0.47
Mn.C	Coarse grain	moderate	0.16 (0.03, 0.29)	0.02	0.17
Mn.C	Coarse grain	high	0.10 (-0.15, 0.35)	0.43	0.67
Mn.C	Dark-Colored vegetables	moderate	0.06 (-0.12, 0.24)	0.50	0.69
Mn.C	Dark-Colored vegetables	high	0.12 (-0.06, 0.30)	0.18	0.47
Mn.C	Fruits	moderate	-0.09 (-0.24, 0.06)	0.25	0.47
Mn.C	Fruits	high	0.03 (-0.13, 0.20)	0.71	0.76
Mn.C	Legumes and soy products	moderate	0.04 (-0.09, 0.18)	0.51	0.69
Mn.C	Legumes and soy products	high	0.11 (-0.06, 0.27)	0.21	0.47
Mn.C	Nuts	moderate	-0.03 (-0.17, 0.10)	0.61	0.71
Mn.C	Nuts	high	-0.05 (-0.29, 0.18)	0.67	0.75
Mn.C	Red meat	moderate	-0.09 (-0.27, 0.09)	0.33	0.58
Mn.C	Red meat	high	0.06 (-0.12, 0.24)	0.52	0.69
Mn.C	Animal offal	moderate	0.13 (-0.02, 0.28)	0.10	0.38
Mn.C	Animal offal	high	-0.01 (-0.33, 0.31)	0.96	0.96
Mn.C	Processed meat	moderate	0.18 (0.04, 0.33)	0.02	0.17
Mn.C	Processed meat	high	-0.16 (-0.52, 0.19)	0.37	0.60
Mn.C	Sugar-sweetened beverages	moderate	0.04 (-0.10, 0.18)	0.56	0.69
Mn.C	Sugar-sweetened beverages	high	0.16 (-0.11, 0.43)	0.24	0.47
Mn.C	Pickled or fried food	moderate	0.19 (0.05, 0.33)	0.01	0.17
Mn.C	Pickled or fried food	high	-0.01 (-0.27, 0.25)	0.94	0.96

Mn.C	Coffee	moderate	0.19 (0.02, 0.36)	0.03	0.17
Mn.C	Coffee	high	0.30 (-0.12, 0.73)	0.16	0.47
Mn.C	Tea	moderate	0.12 (-0.01, 0.25)	0.07	0.34
Mn.C	Tea	high	0.23 (0.02, 0.43)	0.03	0.17
Co.C	White rice	moderate	0.01 (-0.24, 0.27)	0.92	0.99
Co.C	White rice	high	0.05 (-0.21, 0.30)	0.73	0.88
Co.C	Coarse grain	moderate	-0.00 (-0.13, 0.13)	0.99	0.99
Co.C	Coarse grain	high	0.08 (-0.18, 0.33)	0.54	0.76
Co.C	Dark-Colored vegetables	moderate	-0.14 (-0.33, 0.04)	0.12	0.50
Co.C	Dark-Colored vegetables	high	-0.11 (-0.29, 0.07)	0.24	0.52
Co.C	Fruits	moderate	0.08 (-0.07, 0.24)	0.29	0.54
Co.C	Fruits	high	0.12 (-0.05, 0.29)	0.16	0.50
Co.C	Legumes and soy products	moderate	0.10 (-0.04, 0.23)	0.16	0.50
Co.C	Legumes and soy products	high	0.02 (-0.15, 0.19)	0.80	0.93
Co.C	Nuts	moderate	0.12 (-0.01, 0.26)	0.07	0.50
Co.C	Nuts	high	0.24 (0.01, 0.48)	0.04	0.41
Co.C	Red meat	moderate	0.00 (-0.18, 0.19)	0.99	0.99
Co.C	Red meat	high	-0.03 (-0.22, 0.15)	0.72	0.88
Co.C	Animal offal	moderate	0.13 (-0.02, 0.28)	0.10	0.50
Co.C	Animal offal	high	0.23 (-0.10, 0.55)	0.17	0.50
Co.C	Processed meat	moderate	0.10 (-0.06, 0.25)	0.21	0.50
Co.C	Processed meat	high	-0.04 (-0.40, 0.32)	0.83	0.93
Co.C	Sugar-sweetened beverages	moderate	-0.05 (-0.19, 0.08)	0.45	0.75
Co.C	Sugar-sweetened beverages	high	0.09 (-0.18, 0.36)	0.53	0.76
Co.C	Pickled or fried food	moderate	0.09 (-0.04, 0.23)	0.18	0.50
Co.C	Pickled or fried food	high	0.06 (-0.21, 0.33)	0.66	0.88

Co.C	Coffee	moderate	0.19 (0.02, 0.36)	0.03	0.41
Co.C	Coffee	high	0.28 (-0.15, 0.71)	0.20	0.50
Co.C	Tea	moderate	0.15 (0.02, 0.28)	0.03	0.41
Co.C	Tea	high	0.07 (-0.14, 0.28)	0.52	0.76
Ni.C	White rice	moderate	0.10 (-0.16, 0.35)	0.47	0.60
Ni.C	White rice	high	0.27 (0.01, 0.52)	0.04	0.23
Ni.C	Coarse grain	moderate	0.08 (-0.06, 0.21)	0.27	0.49
Ni.C	Coarse grain	high	0.14 (-0.12, 0.39)	0.30	0.49
Ni.C	Dark-Colored vegetables	moderate	0.10 (-0.09, 0.28)	0.31	0.49
Ni.C	Dark-Colored vegetables	high	0.06 (-0.13, 0.24)	0.54	0.66
Ni.C	Fruits	moderate	0.06 (-0.10, 0.22)	0.47	0.60
Ni.C	Fruits	high	0.05 (-0.12, 0.21)	0.58	0.68
Ni.C	Legumes and soy products	moderate	0.10 (-0.04, 0.23)	0.16	0.38
Ni.C	Legumes and soy products	high	0.04 (-0.13, 0.21)	0.64	0.69
Ni.C	Nuts	moderate	0.03 (-0.11, 0.16)	0.72	0.72
Ni.C	Nuts	high	0.19 (-0.05, 0.43)	0.12	0.38
Ni.C	Red meat	moderate	-0.08 (-0.27, 0.10)	0.39	0.54
Ni.C	Red meat	high	-0.04 (-0.23, 0.14)	0.64	0.69
Ni.C	Animal offal	moderate	0.11 (-0.04, 0.27)	0.16	0.38
Ni.C	Animal offal	high	0.23 (-0.10, 0.55)	0.18	0.38
Ni.C	Processed meat	moderate	0.12 (-0.03, 0.27)	0.11	0.38
Ni.C	Processed meat	high	0.17 (-0.19, 0.54)	0.35	0.52
Ni.C	Sugar-sweetened beverages	moderate	0.09 (-0.05, 0.22)	0.23	0.45
Ni.C	Sugar-sweetened beverages	high	0.06 (-0.22, 0.33)	0.68	0.70
Ni.C	Pickled or fried food	moderate	0.18 (0.04, 0.32)	0.01	0.13
Ni.C	Pickled or fried food	high	0.20 (-0.07, 0.47)	0.14	0.38

Ni.C	Coffee	moderate	0.18 (0.01, 0.36)	0.04	0.23
Ni.C	Coffee	high	0.22 (-0.21, 0.66)	0.32	0.49
Ni.C	Tea	moderate	0.11 (-0.02, 0.24)	0.10	0.38
Ni.C	Tea	high	0.27 (0.06, 0.48)	0.01	0.13
Ga.C	White rice	moderate	-0.22 (-0.47, 0.03)	0.09	0.38
Ga.C	White rice	high	-0.07 (-0.32, 0.18)	0.58	0.77
Ga.C	Coarse grain	moderate	0.00 (-0.13, 0.13)	0.96	0.96
Ga.C	Coarse grain	high	-0.15 (-0.40, 0.10)	0.23	0.46
Ga.C	Dark-Colored vegetables	moderate	0.18 (0.00, 0.36)	0.05	0.34
Ga.C	Dark-Colored vegetables	high	0.14 (-0.04, 0.31)	0.12	0.38
Ga.C	Fruits	moderate	0.09 (-0.06, 0.24)	0.26	0.48
Ga.C	Fruits	high	0.23 (0.07, 0.39)	0.01	0.08
Ga.C	Legumes and soy products	moderate	0.09 (-0.04, 0.22)	0.18	0.44
Ga.C	Legumes and soy products	high	0.16 (-0.00, 0.33)	0.05	0.34
Ga.C	Nuts	moderate	-0.04 (-0.17, 0.10)	0.60	0.77
Ga.C	Nuts	high	-0.07 (-0.30, 0.16)	0.56	0.77
Ga.C	Red meat	moderate	0.03 (-0.15, 0.21)	0.73	0.84
Ga.C	Red meat	high	0.12 (-0.06, 0.30)	0.20	0.44
Ga.C	Animal offal	moderate	0.05 (-0.10, 0.20)	0.53	0.77
Ga.C	Animal offal	high	0.02 (-0.30, 0.33)	0.92	0.96
Ga.C	Processed meat	moderate	-0.02 (-0.16, 0.13)	0.81	0.87
Ga.C	Processed meat	high	0.26 (-0.09, 0.61)	0.15	0.42
Ga.C	Sugar-sweetened beverages	moderate	-0.11 (-0.25, 0.02)	0.10	0.38
Ga.C	Sugar-sweetened beverages	high	-0.08 (-0.35, 0.18)	0.55	0.77
Ga.C	Pickled or fried food	moderate	0.03 (-0.11, 0.16)	0.69	0.84
Ga.C	Pickled or fried food	high	0.10 (-0.16, 0.36)	0.44	0.77

Ga.C	Coffee	moderate	0.03 (-0.14, 0.19)	0.75	0.84
Ga.C	Coffee	high	0.40 (-0.02, 0.82)	0.06	0.34
Ga.C	Tea	moderate	0.10 (-0.03, 0.23)	0.12	0.38
Ga.C	Tea	high	0.30 (0.10, 0.50)	0.00	0.08
Rb.C	White rice	moderate	0.06 (-0.19, 0.31)	0.63	0.96
Rb.C	White rice	high	0.10 (-0.14, 0.35)	0.42	0.96
Rb.C	Coarse grain	moderate	0.01 (-0.12, 0.14)	0.91	0.96
Rb.C	Coarse grain	high	-0.15 (-0.40, 0.10)	0.24	0.96
Rb.C	Dark-Colored vegetables	moderate	-0.00 (-0.18, 0.18)	0.97	0.97
Rb.C	Dark-Colored vegetables	high	-0.03 (-0.21, 0.14)	0.70	0.96
Rb.C	Fruits	moderate	0.09 (-0.06, 0.24)	0.25	0.96
Rb.C	Fruits	high	0.12 (-0.04, 0.28)	0.14	0.78
Rb.C	Legumes and soy products	moderate	0.01 (-0.12, 0.14)	0.93	0.96
Rb.C	Legumes and soy products	high	0.01 (-0.15, 0.18)	0.88	0.96
Rb.C	Nuts	moderate	-0.05 (-0.19, 0.08)	0.44	0.96
Rb.C	Nuts	high	-0.03 (-0.27, 0.20)	0.78	0.96
Rb.C	Red meat	moderate	-0.04 (-0.22, 0.14)	0.67	0.96
Rb.C	Red meat	high	0.09 (-0.09, 0.27)	0.32	0.96
Rb.C	Animal offal	moderate	-0.04 (-0.19, 0.11)	0.57	0.96
Rb.C	Animal offal	high	-0.14 (-0.45, 0.18)	0.40	0.96
Rb.C	Processed meat	moderate	-0.03 (-0.17, 0.12)	0.73	0.96
Rb.C	Processed meat	high	-0.12 (-0.47, 0.24)	0.52	0.96
Rb.C	Sugar-sweetened beverages	moderate	-0.10 (-0.24, 0.03)	0.13	0.78
Rb.C	Sugar-sweetened beverages	high	-0.09 (-0.36, 0.18)	0.50	0.96
Rb.C	Pickled or fried food	moderate	-0.07 (-0.21, 0.07)	0.32	0.96
Rb.C	Pickled or fried food	high	-0.02 (-0.29, 0.24)	0.86	0.96

Rb.C	Coffee	moderate	-0.01 (-0.18, 0.16)	0.92	0.96
Rb.C	Coffee	high	0.37 (-0.05, 0.79)	0.09	0.78
Rb.C	Tea	moderate	0.10 (-0.03, 0.23)	0.12	0.78
Rb.C	Tea	high	0.41 (0.21, 0.62)	0.0000631620282579003	0.00
Sn.C	White rice	moderate	0.14 (-0.12, 0.40)	0.29	0.53
Sn.C	White rice	high	0.12 (-0.13, 0.38)	0.35	0.54
Sn.C	Coarse grain	moderate	0.25 (0.12, 0.38)	0.00	0.01
Sn.C	Coarse grain	high	0.16 (-0.10, 0.41)	0.23	0.53
Sn.C	Dark-Colored vegetables	moderate	0.15 (-0.04, 0.33)	0.11	0.47
Sn.C	Dark-Colored vegetables	high	0.06 (-0.12, 0.24)	0.50	0.64
Sn.C	Fruits	moderate	0.08 (-0.07, 0.24)	0.30	0.53
Sn.C	Fruits	high	0.14 (-0.03, 0.30)	0.11	0.47
Sn.C	Legumes and soy products	moderate	0.13 (-0.00, 0.26)	0.06	0.47
Sn.C	Legumes and soy products	high	0.09 (-0.08, 0.26)	0.30	0.53
Sn.C	Nuts	moderate	0.03 (-0.11, 0.17)	0.69	0.74
Sn.C	Nuts	high	-0.05 (-0.29, 0.19)	0.71	0.74
Sn.C	Red meat	moderate	0.17 (-0.01, 0.36)	0.07	0.47
Sn.C	Red meat	high	0.19 (0.00, 0.37)	0.05	0.47
Sn.C	Animal offal	moderate	0.12 (-0.04, 0.27)	0.13	0.47
Sn.C	Animal offal	high	-0.07 (-0.39, 0.26)	0.69	0.74
Sn.C	Processed meat	moderate	-0.00 (-0.16, 0.15)	0.95	0.95
Sn.C	Processed meat	high	-0.13 (-0.50, 0.23)	0.47	0.63
Sn.C	Sugar-sweetened beverages	moderate	0.09 (-0.05, 0.23)	0.21	0.53
Sn.C	Sugar-sweetened beverages	high	0.13 (-0.15, 0.40)	0.36	0.54
Sn.C	Pickled or fried food	moderate	0.09 (-0.05, 0.23)	0.22	0.53
Sn.C	Pickled or fried food	high	0.09 (-0.18, 0.36)	0.53	0.64

Sn.C	Coffee	moderate	0.12 (-0.05, 0.29)	0.17	0.53
Sn.C	Coffee	high	-0.18 (-0.61, 0.26)	0.42	0.59
Sn.C	Tea	moderate	-0.07 (-0.20, 0.07)	0.33	0.54
Sn.C	Tea	high	0.12 (-0.09, 0.33)	0.27	0.53
Sr.C	White rice	moderate	-0.07 (-0.32, 0.18)	0.58	0.70
Sr.C	White rice	high	-0.18 (-0.43, 0.06)	0.14	0.40
Sr.C	Coarse grain	moderate	0.09 (-0.04, 0.22)	0.17	0.42
Sr.C	Coarse grain	high	0.01 (-0.24, 0.26)	0.94	0.94
Sr.C	Dark-Colored vegetables	moderate	0.02 (-0.16, 0.20)	0.82	0.92
Sr.C	Dark-Colored vegetables	high	0.08 (-0.10, 0.25)	0.39	0.64
Sr.C	Fruits	moderate	-0.03 (-0.18, 0.12)	0.72	0.84
Sr.C	Fruits	high	-0.16 (-0.32, 0.00)	0.05	0.28
Sr.C	Legumes and soy products	moderate	0.12 (-0.01, 0.25)	0.07	0.30
Sr.C	Legumes and soy products	high	0.11 (-0.06, 0.28)	0.19	0.42
Sr.C	Nuts	moderate	0.04 (-0.09, 0.17)	0.55	0.70
Sr.C	Nuts	high	0.14 (-0.10, 0.37)	0.26	0.51
Sr.C	Red meat	moderate	-0.09 (-0.27, 0.09)	0.31	0.59
Sr.C	Red meat	high	-0.01 (-0.19, 0.17)	0.92	0.94
Sr.C	Animal offal	moderate	0.18 (0.03, 0.33)	0.02	0.11
Sr.C	Animal offal	high	0.11 (-0.20, 0.43)	0.48	0.68
Sr.C	Processed meat	moderate	0.11 (-0.04, 0.26)	0.14	0.40
Sr.C	Processed meat	high	0.16 (-0.19, 0.52)	0.37	0.64
Sr.C	Sugar-sweetened beverages	moderate	0.01 (-0.12, 0.15)	0.86	0.93
Sr.C	Sugar-sweetened beverages	high	0.11 (-0.16, 0.37)	0.43	0.66
Sr.C	Pickled or fried food	moderate	0.09 (-0.05, 0.23)	0.20	0.42
Sr.C	Pickled or fried food	high	0.10 (-0.16, 0.36)	0.45	0.66

Sr.C	Coffee	moderate	0.15 (-0.02, 0.31)	0.09	0.31
Sr.C	Coffee	high	0.38 (-0.04, 0.80)	0.08	0.30
Sr.C	Tea	moderate	0.16 (0.03, 0.29)	0.01	0.11
Sr.C	Tea	high	0.27 (0.07, 0.48)	0.01	0.11
Ce.C	White rice	moderate	-0.21 (-0.47, 0.04)	0.10	0.41
Ce.C	White rice	high	-0.10 (-0.35, 0.16)	0.46	0.64
Ce.C	Coarse grain	moderate	0.02 (-0.11, 0.16)	0.71	0.85
Ce.C	Coarse grain	high	-0.14 (-0.39, 0.11)	0.28	0.53
Ce.C	Dark-Colored vegetables	moderate	0.25 (0.07, 0.44)	0.01	0.09
Ce.C	Dark-Colored vegetables	high	0.18 (-0.00, 0.36)	0.05	0.36
Ce.C	Fruits	moderate	0.11 (-0.05, 0.26)	0.18	0.53
Ce.C	Fruits	high	0.24 (0.08, 0.41)	0.00	0.09
Ce.C	Legumes and soy products	moderate	0.02 (-0.11, 0.16)	0.73	0.85
Ce.C	Legumes and soy products	high	0.10 (-0.07, 0.27)	0.24	0.53
Ce.C	Nuts	moderate	0.03 (-0.11, 0.16)	0.69	0.85
Ce.C	Nuts	high	-0.15 (-0.39, 0.09)	0.21	0.53
Ce.C	Red meat	moderate	0.12 (-0.06, 0.31)	0.19	0.53
Ce.C	Red meat	high	0.23 (0.05, 0.42)	0.01	0.13
Ce.C	Animal offal	moderate	-0.01 (-0.16, 0.14)	0.90	0.90
Ce.C	Animal offal	high	0.03 (-0.30, 0.35)	0.86	0.90
Ce.C	Processed meat	moderate	-0.06 (-0.21, 0.09)	0.42	0.64
Ce.C	Processed meat	high	0.20 (-0.16, 0.56)	0.28	0.53
Ce.C	Sugar-sweetened beverages	moderate	-0.07 (-0.21, 0.07)	0.32	0.53
Ce.C	Sugar-sweetened beverages	high	-0.04 (-0.31, 0.23)	0.78	0.86
Ce.C	Pickled or fried food	moderate	0.07 (-0.07, 0.21)	0.32	0.53
Ce.C	Pickled or fried food	high	0.16 (-0.11, 0.43)	0.24	0.53

Ce.C	Coffee	moderate	-0.02 (-0.19, 0.15)	0.80	0.86
Ce.C	Coffee	high	0.39 (-0.04, 0.82)	0.08	0.37
Ce.C	Tea	moderate	0.02 (-0.11, 0.15)	0.72	0.85
Ce.C	Tea	high	0.19 (-0.02, 0.39)	0.08	0.37
Cu.C	White rice	moderate	-0.03 (-0.28, 0.21)	0.78	0.96
Cu.C	White rice	high	-0.12 (-0.35, 0.12)	0.34	0.96
Cu.C	Coarse grain	moderate	0.01 (-0.12, 0.13)	0.94	0.98
Cu.C	Coarse grain	high	-0.17 (-0.41, 0.07)	0.17	0.96
Cu.C	Dark-Colored vegetables	moderate	0.05 (-0.13, 0.22)	0.60	0.96
Cu.C	Dark-Colored vegetables	high	-0.04 (-0.21, 0.13)	0.68	0.96
Cu.C	Fruits	moderate	-0.02 (-0.17, 0.13)	0.79	0.96
Cu.C	Fruits	high	-0.07 (-0.23, 0.09)	0.38	0.96
Cu.C	Legumes and soy products	moderate	-0.02 (-0.14, 0.11)	0.79	0.96
Cu.C	Legumes and soy products	high	-0.03 (-0.19, 0.13)	0.70	0.96
Cu.C	Nuts	moderate	-0.03 (-0.16, 0.10)	0.61	0.96
Cu.C	Nuts	high	-0.00 (-0.23, 0.22)	1.00	1.00
Cu.C	Red meat	moderate	-0.05 (-0.23, 0.12)	0.55	0.96
Cu.C	Red meat	high	-0.05 (-0.23, 0.12)	0.55	0.96
Cu.C	Animal offal	moderate	-0.04 (-0.18, 0.11)	0.62	0.96
Cu.C	Animal offal	high	-0.02 (-0.33, 0.28)	0.88	0.98
Cu.C	Processed meat	moderate	0.06 (-0.09, 0.20)	0.44	0.96
Cu.C	Processed meat	high	-0.17 (-0.51, 0.17)	0.32	0.96
Cu.C	Sugar-sweetened beverages	moderate	-0.10 (-0.23, 0.03)	0.14	0.96
Cu.C	Sugar-sweetened beverages	high	-0.21 (-0.47, 0.04)	0.10	0.96
Cu.C	Pickled or fried food	moderate	-0.06 (-0.19, 0.07)	0.37	0.96
Cu.C	Pickled or fried food	high	-0.17 (-0.42, 0.09)	0.20	0.96

Cu.C	Coffee	moderate	-0.09 (-0.25, 0.07)	0.27	0.96
Cu.C	Coffee	high	-0.20 (-0.61, 0.20)	0.32	0.96
Cu.C	Tea	moderate	0.04 (-0.09, 0.16)	0.56	0.96
Cu.C	Tea	high	0.02 (-0.18, 0.22)	0.86	0.98
Ag.C	White rice	moderate	0.10 (-0.15, 0.36)	0.43	0.87
Ag.C	White rice	high	0.05 (-0.21, 0.30)	0.71	0.87
Ag.C	Coarse grain	moderate	0.14 (0.00, 0.27)	0.04	0.75
Ag.C	Coarse grain	high	0.13 (-0.13, 0.38)	0.33	0.87
Ag.C	Dark-Colored vegetables	moderate	0.05 (-0.13, 0.24)	0.57	0.87
Ag.C	Dark-Colored vegetables	high	0.04 (-0.14, 0.22)	0.66	0.87
Ag.C	Fruits	moderate	-0.09 (-0.25, 0.06)	0.25	0.87
Ag.C	Fruits	high	-0.07 (-0.24, 0.10)	0.42	0.87
Ag.C	Legumes and soy products	moderate	0.01 (-0.12, 0.15)	0.87	0.92
Ag.C	Legumes and soy products	high	0.03 (-0.14, 0.20)	0.71	0.87
Ag.C	Nuts	moderate	0.04 (-0.10, 0.17)	0.62	0.87
Ag.C	Nuts	high	0.04 (-0.20, 0.28)	0.72	0.87
Ag.C	Red meat	moderate	0.05 (-0.14, 0.24)	0.59	0.87
Ag.C	Red meat	high	0.17 (-0.02, 0.35)	0.08	0.75
Ag.C	Animal offal	moderate	0.13 (-0.03, 0.28)	0.11	0.75
Ag.C	Animal offal	high	-0.02 (-0.35, 0.31)	0.91	0.92
Ag.C	Processed meat	moderate	0.02 (-0.13, 0.17)	0.78	0.87
Ag.C	Processed meat	high	0.18 (-0.19, 0.54)	0.35	0.87
Ag.C	Sugar-sweetened beverages	moderate	0.05 (-0.09, 0.19)	0.50	0.87
Ag.C	Sugar-sweetened beverages	high	0.15 (-0.13, 0.42)	0.30	0.87
Ag.C	Pickled or fried food	moderate	0.03 (-0.10, 0.17)	0.62	0.87
Ag.C	Pickled or fried food	high	0.24 (-0.03, 0.51)	0.08	0.75

Ag.C	Coffee	moderate	0.03 (-0.14, 0.20)	0.72	0.87
Ag.C	Coffee	high	0.32 (-0.11, 0.76)	0.14	0.80
Ag.C	Tea	moderate	0.04 (-0.09, 0.17)	0.54	0.87
Ag.C	Tea	high	-0.01 (-0.22, 0.20)	0.92	0.92
Cd.C	White rice	moderate	-0.04 (-0.30, 0.22)	0.76	0.97
Cd.C	White rice	high	0.09 (-0.17, 0.34)	0.50	0.97
Cd.C	Coarse grain	moderate	0.07 (-0.06, 0.21)	0.28	0.97
Cd.C	Coarse grain	high	0.09 (-0.17, 0.35)	0.49	0.97
Cd.C	Dark-Colored vegetables	moderate	-0.01 (-0.19, 0.18)	0.93	0.97
Cd.C	Dark-Colored vegetables	high	0.01 (-0.17, 0.19)	0.90	0.97
Cd.C	Fruits	moderate	-0.02 (-0.18, 0.14)	0.80	0.97
Cd.C	Fruits	high	-0.01 (-0.17, 0.16)	0.94	0.97
Cd.C	Legumes and soy products	moderate	0.11 (-0.03, 0.24)	0.12	0.83
Cd.C	Legumes and soy products	high	-0.02 (-0.19, 0.15)	0.79	0.97
Cd.C	Nuts	moderate	-0.00 (-0.14, 0.13)	0.96	0.97
Cd.C	Nuts	high	0.09 (-0.15, 0.33)	0.45	0.97
Cd.C	Red meat	moderate	0.16 (-0.02, 0.35)	0.09	0.83
Cd.C	Red meat	high	0.04 (-0.15, 0.22)	0.70	0.97
Cd.C	Animal offal	moderate	0.09 (-0.07, 0.24)	0.26	0.97
Cd.C	Animal offal	high	0.03 (-0.30, 0.36)	0.87	0.97
Cd.C	Processed meat	moderate	0.13 (-0.03, 0.28)	0.10	0.83
Cd.C	Processed meat	high	-0.19 (-0.55, 0.18)	0.32	0.97
Cd.C	Sugar-sweetened beverages	moderate	0.05 (-0.09, 0.19)	0.49	0.97
Cd.C	Sugar-sweetened beverages	high	-0.03 (-0.31, 0.24)	0.81	0.97
Cd.C	Pickled or fried food	moderate	0.06 (-0.08, 0.20)	0.39	0.97
Cd.C	Pickled or fried food	high	-0.07 (-0.34, 0.20)	0.63	0.97

Cd.C	Coffee	moderate	0.10 (-0.08, 0.27)	0.27	0.97
Cd.C	Coffee	high	-0.01 (-0.44, 0.43)	0.97	0.97
Cd.C	Tea	moderate	-0.01 (-0.14, 0.12)	0.90	0.97
Cd.C	Tea	high	0.12 (-0.09, 0.33)	0.28	0.97
Ba.C	White rice	moderate	-0.09 (-0.35, 0.17)	0.48	0.67
Ba.C	White rice	high	0.02 (-0.24, 0.27)	0.90	0.93
Ba.C	Coarse grain	moderate	0.04 (-0.09, 0.18)	0.56	0.71
Ba.C	Coarse grain	high	0.05 (-0.21, 0.31)	0.69	0.77
Ba.C	Dark-Colored vegetables	moderate	0.08 (-0.10, 0.27)	0.38	0.60
Ba.C	Dark-Colored vegetables	high	0.11 (-0.07, 0.29)	0.24	0.47
Ba.C	Fruits	moderate	0.09 (-0.07, 0.25)	0.25	0.47
Ba.C	Fruits	high	0.15 (-0.02, 0.32)	0.08	0.30
Ba.C	Legumes and soy products	moderate	0.01 (-0.13, 0.14)	0.93	0.93
Ba.C	Legumes and soy products	high	0.13 (-0.04, 0.30)	0.15	0.40
Ba.C	Nuts	moderate	-0.03 (-0.17, 0.11)	0.68	0.77
Ba.C	Nuts	high	0.06 (-0.18, 0.31)	0.60	0.73
Ba.C	Red meat	moderate	0.06 (-0.12, 0.25)	0.51	0.68
Ba.C	Red meat	high	0.13 (-0.06, 0.32)	0.17	0.41
Ba.C	Animal offal	moderate	0.15 (-0.00, 0.31)	0.05	0.30
Ba.C	Animal offal	high	0.24 (-0.09, 0.57)	0.16	0.40
Ba.C	Processed meat	moderate	0.07 (-0.08, 0.22)	0.35	0.60
Ba.C	Processed meat	high	0.38 (0.02, 0.75)	0.04	0.30
Ba.C	Sugar-sweetened beverages	moderate	-0.05 (-0.19, 0.09)	0.48	0.67
Ba.C	Sugar-sweetened beverages	high	0.18 (-0.10, 0.45)	0.20	0.43
Ba.C	Pickled or fried food	moderate	0.12 (-0.02, 0.26)	0.09	0.30
Ba.C	Pickled or fried food	high	0.24 (-0.03, 0.51)	0.08	0.30

Ba.C	Coffee	moderate	0.16 (-0.01, 0.34)	0.07	0.30
Ba.C	Coffee	high	0.47 (0.04, 0.91)	0.03	0.30
Ba.C	Tea	moderate	0.02 (-0.11, 0.16)	0.72	0.77
Ba.C	Tea	high	0.23 (0.02, 0.44)	0.03	0.30
Se.C	White rice	moderate	0.15 (-0.10, 0.41)	0.23	0.69
Se.C	White rice	high	0.20 (-0.04, 0.45)	0.11	0.50
Se.C	Coarse grain	moderate	-0.06 (-0.19, 0.07)	0.39	0.73
Se.C	Coarse grain	high	-0.21 (-0.46, 0.04)	0.11	0.50
Se.C	Dark-Colored vegetables	moderate	0.04 (-0.14, 0.22)	0.70	0.80
Se.C	Dark-Colored vegetables	high	0.07 (-0.11, 0.25)	0.44	0.73
Se.C	Fruits	moderate	-0.03 (-0.19, 0.12)	0.68	0.80
Se.C	Fruits	high	0.07 (-0.09, 0.24)	0.37	0.73
Se.C	Legumes and soy products	moderate	-0.02 (-0.15, 0.11)	0.76	0.80
Se.C	Legumes and soy products	high	-0.00 (-0.17, 0.16)	0.96	0.96
Se.C	Nuts	moderate	-0.08 (-0.21, 0.06)	0.27	0.69
Se.C	Nuts	high	-0.23 (-0.46, 0.00)	0.05	0.40
Se.C	Red meat	moderate	0.03 (-0.15, 0.22)	0.71	0.80
Se.C	Red meat	high	0.18 (-0.00, 0.36)	0.05	0.40
Se.C	Animal offal	moderate	0.04 (-0.11, 0.19)	0.59	0.79
Se.C	Animal offal	high	-0.05 (-0.37, 0.27)	0.77	0.80
Se.C	Processed meat	moderate	-0.04 (-0.19, 0.11)	0.59	0.79
Se.C	Processed meat	high	-0.21 (-0.57, 0.15)	0.25	0.69
Se.C	Sugar-sweetened beverages	moderate	-0.10 (-0.23, 0.04)	0.15	0.59
Se.C	Sugar-sweetened beverages	high	-0.13 (-0.40, 0.14)	0.35	0.73
Se.C	Pickled or fried food	moderate	-0.05 (-0.19, 0.09)	0.48	0.75
Se.C	Pickled or fried food	high	-0.27 (-0.53, -0.00)	0.05	0.40

Se.C	Coffee	moderate	-0.07 (-0.24, 0.10)	0.44	0.73
Se.C	Coffee	high	-0.12 (-0.54, 0.30)	0.58	0.79
Se.C	Tea	moderate	0.07 (-0.06, 0.19)	0.31	0.73
Se.C	Tea	high	-0.12 (-0.33, 0.08)	0.25	0.69
Zn.C	White rice	moderate	0.14 (-0.12, 0.40)	0.29	0.66
Zn.C	White rice	high	0.18 (-0.07, 0.44)	0.15	0.66
Zn.C	Coarse grain	moderate	0.11 (-0.02, 0.25)	0.10	0.66
Zn.C	Coarse grain	high	0.13 (-0.13, 0.38)	0.32	0.66
Zn.C	Dark-Colored vegetables	moderate	0.07 (-0.11, 0.25)	0.45	0.66
Zn.C	Dark-Colored vegetables	high	0.09 (-0.09, 0.27)	0.34	0.66
Zn.C	Fruits	moderate	-0.01 (-0.16, 0.15)	0.90	0.90
Zn.C	Fruits	high	0.05 (-0.12, 0.22)	0.56	0.71
Zn.C	Legumes and soy products	moderate	0.08 (-0.06, 0.21)	0.26	0.66
Zn.C	Legumes and soy products	high	0.02 (-0.15, 0.18)	0.85	0.90
Zn.C	Nuts	moderate	-0.01 (-0.15, 0.13)	0.88	0.90
Zn.C	Nuts	high	0.09 (-0.15, 0.33)	0.47	0.66
Zn.C	Red meat	moderate	-0.06 (-0.24, 0.13)	0.55	0.71
Zn.C	Red meat	high	0.04 (-0.15, 0.22)	0.71	0.86
Zn.C	Animal offal	moderate	0.11 (-0.04, 0.26)	0.16	0.66
Zn.C	Animal offal	high	0.03 (-0.29, 0.36)	0.84	0.90
Zn.C	Processed meat	moderate	0.19 (0.04, 0.34)	0.01	0.31
Zn.C	Processed meat	high	-0.35 (-0.71, 0.01)	0.06	0.56
Zn.C	Sugar-sweetened beverages	moderate	0.02 (-0.12, 0.16)	0.77	0.89
Zn.C	Sugar-sweetened beverages	high	0.10 (-0.17, 0.37)	0.46	0.66
Zn.C	Pickled or fried food	moderate	0.09 (-0.05, 0.23)	0.20	0.66
Zn.C	Pickled or fried food	high	-0.11 (-0.38, 0.16)	0.43	0.66

Zn.C	Coffee	moderate	0.07 (-0.10, 0.25)	0.40	0.66
Zn.C	Coffee	high	0.19 (-0.24, 0.63)	0.37	0.66
Zn.C	Tea	moderate	0.09 (-0.04, 0.22)	0.19	0.66
Zn.C	Tea	high	0.20 (-0.01, 0.41)	0.06	0.56
Tl.C	White rice	moderate	-0.13 (-0.39, 0.13)	0.32	0.84
Tl.C	White rice	high	-0.17 (-0.42, 0.09)	0.20	0.84
Tl.C	Coarse grain	moderate	-0.04 (-0.18, 0.09)	0.52	0.84
Tl.C	Coarse grain	high	0.10 (-0.15, 0.36)	0.43	0.84
Tl.C	Dark-Colored vegetables	moderate	-0.05 (-0.24, 0.13)	0.58	0.84
Tl.C	Dark-Colored vegetables	high	-0.04 (-0.22, 0.14)	0.69	0.84
Tl.C	Fruits	moderate	-0.05 (-0.20, 0.11)	0.56	0.84
Tl.C	Fruits	high	-0.02 (-0.19, 0.15)	0.83	0.86
Tl.C	Legumes and soy products	moderate	-0.04 (-0.18, 0.09)	0.53	0.84
Tl.C	Legumes and soy products	high	0.02 (-0.15, 0.19)	0.83	0.86
Tl.C	Nuts	moderate	-0.15 (-0.28, -0.01)	0.04	0.35
Tl.C	Nuts	high	0.03 (-0.21, 0.27)	0.81	0.86
Tl.C	Red meat	moderate	-0.15 (-0.33, 0.04)	0.12	0.84
Tl.C	Red meat	high	-0.21 (-0.39, -0.02)	0.03	0.35
Tl.C	Animal offal	moderate	0.07 (-0.08, 0.23)	0.34	0.84
Tl.C	Animal offal	high	0.11 (-0.22, 0.44)	0.51	0.84
Tl.C	Processed meat	moderate	0.01 (-0.14, 0.16)	0.87	0.87
Tl.C	Processed meat	high	0.07 (-0.30, 0.43)	0.72	0.84
Tl.C	Sugar-sweetened beverages	moderate	-0.15 (-0.29, -0.02)	0.03	0.35
Tl.C	Sugar-sweetened beverages	high	0.05 (-0.22, 0.32)	0.72	0.84
Tl.C	Pickled or fried food	moderate	0.03 (-0.11, 0.17)	0.66	0.84
Tl.C	Pickled or fried food	high	-0.10 (-0.37, 0.17)	0.45	0.84

Tl.C	Coffee	moderate	0.04 (-0.14, 0.21)	0.68	0.84
Tl.C	Coffee	high	0.09 (-0.35, 0.52)	0.69	0.84
Tl.C	Tea	moderate	-0.03 (-0.16, 0.10)	0.62	0.84
Tl.C	Tea	high	-0.07 (-0.28, 0.14)	0.50	0.84
Mo.C	White rice	moderate	0.00 (-0.25, 0.26)	0.97	0.97
Mo.C	White rice	high	0.25 (-0.00, 0.50)	0.05	0.76
Mo.C	Coarse grain	moderate	-0.01 (-0.15, 0.12)	0.85	0.97
Mo.C	Coarse grain	high	0.02 (-0.23, 0.28)	0.86	0.97
Mo.C	Dark-Colored vegetables	moderate	-0.01 (-0.19, 0.18)	0.95	0.97
Mo.C	Dark-Colored vegetables	high	-0.01 (-0.20, 0.17)	0.87	0.97
Mo.C	Fruits	moderate	-0.04 (-0.20, 0.12)	0.61	0.90
Mo.C	Fruits	high	-0.09 (-0.26, 0.07)	0.27	0.90
Mo.C	Legumes and soy products	moderate	0.04 (-0.09, 0.18)	0.53	0.90
Mo.C	Legumes and soy products	high	0.21 (0.05, 0.38)	0.01	0.35
Mo.C	Nuts	moderate	-0.06 (-0.20, 0.08)	0.38	0.90
Mo.C	Nuts	high	0.14 (-0.10, 0.37)	0.26	0.90
Mo.C	Red meat	moderate	-0.09 (-0.28, 0.09)	0.32	0.90
Mo.C	Red meat	high	0.06 (-0.12, 0.25)	0.50	0.90
Mo.C	Animal offal	moderate	-0.04 (-0.20, 0.11)	0.59	0.90
Mo.C	Animal offal	high	-0.02 (-0.35, 0.30)	0.90	0.97
Mo.C	Processed meat	moderate	-0.02 (-0.17, 0.13)	0.83	0.97
Mo.C	Processed meat	high	-0.06 (-0.43, 0.30)	0.73	0.97
Mo.C	Sugar-sweetened beverages	moderate	-0.09 (-0.23, 0.05)	0.21	0.90
Mo.C	Sugar-sweetened beverages	high	0.09 (-0.18, 0.36)	0.51	0.90
Mo.C	Pickled or fried food	moderate	0.08 (-0.06, 0.22)	0.24	0.90
Mo.C	Pickled or fried food	high	0.09 (-0.18, 0.36)	0.52	0.90

Mo.C	Coffee	moderate	0.05 (-0.12, 0.22)	0.55	0.90
Mo.C	Coffee	high	0.15 (-0.28, 0.58)	0.49	0.90
Mo.C	Tea	moderate	-0.09 (-0.22, 0.04)	0.16	0.90
Mo.C	Tea	high	0.07 (-0.14, 0.28)	0.51	0.90

Table S8. Association between dietary intake and trace elements concentrations among 1,066 females.

Trace elements-Female	Dietary	Beta (95% CI)	P-value	P-FDR
Sn-F	Coarse grain	0.16(0.05,0.26)	0.00	0.65
Mo-F	White rice	0.07(0.02,0.11)	0.00	0.65
Cu-F	Sugar-sweetened beverages	-0.05(-0.08,-0.01)	0.01	0.88
Ce-F	Red meat	0.05(0.01,0.1)	0.02	0.88
Tl-F	Sugar-sweetened beverages	-0.11(-0.21,-0.02)	0.02	0.88
Cu-F	Coarse grain	-0.04(-0.07,0)	0.03	0.88
Ni-F	White rice	0.08(0.01,0.15)	0.03	0.88
Sn-F	Red meat	0.1(0.01,0.18)	0.03	0.88
Al-F	Coffee	0.12(0,0.24)	0.04	0.88
Ag-F	Red meat	0.06(0,0.13)	0.05	0.88
Se-F	Coarse grain	-0.03(-0.05,0)	0.05	0.88
Rb-F	Red meat	0.02(0,0.05)	0.05	0.88
Ni-F	Tea	0.07(0,0.14)	0.06	0.88
Sr-F	Tea	0.04(0,0.08)	0.06	0.88
Se-F	Red meat	0.02(0,0.04)	0.06	0.88
Ce-F	Animal offal	-0.06(-0.13,0)	0.06	0.88

Co-F	Coffee	0.11(0,0.22)	0.06	0.88
Cd-F	White rice	0.12(-0.01,0.25)	0.06	0.88
Ga-F	Red meat	0.04(0,0.09)	0.06	0.88
Cu-F	White rice	-0.03(-0.06,0)	0.08	0.88
Sn-F	Pickled or fried food	0.1(-0.01,0.22)	0.09	0.88
Mn-F	Nuts	-0.06(-0.14,0.01)	0.09	0.88
Ba-F	Tea	0.04(-0.01,0.1)	0.10	0.88
Sr-F	Fruits	-0.03(-0.06,0.01)	0.10	0.88
Rb-F	Tea	0.02(0,0.05)	0.10	0.88
Tl-F	Pickled or fried food	-0.07(-0.17,0.02)	0.12	0.88
Ag-F	White rice	-0.06(-0.14,0.02)	0.13	0.88
Mo-F	Fruits	-0.03(-0.07,0.01)	0.13	0.88
Mn-F	Coffee	0.08(-0.03,0.18)	0.14	0.88
Mn-F	Coarse grain	0.06(-0.02,0.13)	0.14	0.88
Sr-F	Animal offal	0.04(-0.01,0.09)	0.14	0.88
Tl-F	Processed meat	-0.08(-0.19,0.03)	0.14	0.88
Zn-F	Tea	0.07(-0.02,0.16)	0.15	0.88
Sn-F	Sugar-sweetened beverages	0.09(-0.03,0.21)	0.15	0.88
Zn-F	Coarse grain	0.06(-0.02,0.15)	0.15	0.88
Fe-F	Sugar-sweetened beverages	-0.05(-0.11,0.02)	0.16	0.88
Cd-F	Red meat	-0.07(-0.18,0.03)	0.16	0.88
Ba-F	Red meat	0.03(-0.01,0.07)	0.16	0.88
V-F	Fruits	-0.06(-0.14,0.02)	0.16	0.88
Se-F	Nuts	-0.02(-0.05,0.01)	0.17	0.88
Sr-F	Coffee	0.04(-0.02,0.09)	0.17	0.88
Cd-F	Pickled or fried food	-0.1(-0.24,0.04)	0.17	0.88

Al-F	Dark-colored vegetables	0.05(-0.02,0.12)	0.18	0.88
Tl-F	Red meat	-0.05(-0.12,0.02)	0.18	0.88
Ag-F	Pickled or fried food	0.06(-0.03,0.14)	0.18	0.88
Ce-F	Processed meat	-0.05(-0.11,0.02)	0.18	0.88
Ce-F	Fruits	0.03(-0.01,0.07)	0.19	0.88
V-F	Coarse grain	0.06(-0.03,0.16)	0.19	0.88
V-F	Pickled or fried food	-0.07(-0.17,0.03)	0.19	0.88
V-F	Nuts	-0.06(-0.15,0.03)	0.19	0.88
Sr-F	White rice	-0.03(-0.07,0.01)	0.20	0.88
Ba-F	Coffee	0.05(-0.02,0.12)	0.21	0.88
Ag-F	Legumes and soy products	-0.04(-0.1,0.02)	0.21	0.88
Al-F	Coarse grain	0.05(-0.03,0.14)	0.21	0.88
Sr-F	Processed meat	0.03(-0.02,0.08)	0.21	0.88
Sr-F	Red meat	-0.02(-0.05,0.01)	0.21	0.88
Rb-F	Fruits	0.01(-0.01,0.04)	0.21	0.88
Al-F	Red meat	0.04(-0.03,0.11)	0.21	0.88
Ga-F	Animal offal	-0.04(-0.11,0.03)	0.22	0.88
Zn-F	Animal offal	0.07(-0.04,0.17)	0.22	0.88
Rb-F	Coffee	0.02(-0.01,0.06)	0.22	0.88
Co-F	Coarse grain	-0.05(-0.13,0.03)	0.23	0.88
Ba-F	White rice	0.03(-0.02,0.08)	0.23	0.88
Cu-F	Coffee	-0.03(-0.07,0.02)	0.23	0.88
Ga-F	Coarse grain	-0.03(-0.09,0.02)	0.23	0.88
Fe-F	White rice	0.04(-0.02,0.1)	0.23	0.88
Ba-F	Pickled or fried food	0.03(-0.02,0.09)	0.24	0.88
Mn-F	Dark-colored vegetables	0.04(-0.02,0.1)	0.24	0.88

Al-F	Tea	0.05(-0.04,0.14)	0.24	0.88
Be-F	Sugar-sweetened beverages	-0.1(-0.28,0.07)	0.24	0.88
Al-F	Fruits	-0.04(-0.11,0.03)	0.24	0.88
Zn-F	Pickled or fried food	-0.06(-0.15,0.04)	0.24	0.88
Li-F	Dark-colored vegetables	-0.08(-0.21,0.05)	0.25	0.88
Sn-F	White rice	0.06(-0.05,0.17)	0.25	0.88
Be-F	Nuts	-0.09(-0.24,0.06)	0.26	0.88
Sn-F	Legumes and soy products	0.05(-0.04,0.14)	0.26	0.88
Cu-F	Processed meat	-0.02(-0.07,0.02)	0.26	0.88
Sr-F	Dark-colored vegetables	0.02(-0.01,0.05)	0.26	0.88
Cu-F	Pickled or fried food	-0.02(-0.06,0.02)	0.26	0.88
Li-F	Tea	0.09(-0.07,0.26)	0.26	0.88
Ni-F	Pickled or fried food	0.04(-0.03,0.12)	0.27	0.88
V-F	Red meat	-0.04(-0.12,0.04)	0.28	0.88
Sr-F	Pickled or fried food	0.02(-0.02,0.07)	0.28	0.88
Li-F	Animal offal	0.11(-0.09,0.3)	0.28	0.88
Mo-F	Nuts	-0.02(-0.07,0.02)	0.28	0.88
Ga-F	Tea	0.03(-0.03,0.09)	0.29	0.88
V-F	Animal offal	0.06(-0.05,0.18)	0.29	0.88
Rb-F	Coarse grain	-0.01(-0.04,0.01)	0.29	0.88
Sn-F	Fruits	0.05(-0.04,0.14)	0.30	0.88
V-F	Sugar-sweetened beverages	-0.06(-0.16,0.05)	0.30	0.88
Ni-F	Coffee	0.05(-0.05,0.15)	0.30	0.88
Co-F	Dark-colored vegetables	-0.04(-0.11,0.03)	0.30	0.88
Tl-F	Dark-colored vegetables	0.04(-0.03,0.11)	0.31	0.88
Cu-F	Animal offal	-0.02(-0.06,0.02)	0.31	0.88

Ga-F	Dark-colored vegetables	0.02(-0.02,0.07)	0.31	0.88
Sn-F	Tea	0.06(-0.05,0.17)	0.32	0.88
Mn-F	Tea	0.04(-0.04,0.12)	0.32	0.88
Li-F	Coarse grain	0.08(-0.08,0.23)	0.32	0.89
Ag-F	Processed meat	-0.05(-0.15,0.05)	0.33	0.89
Ag-F	Fruits	-0.03(-0.1,0.03)	0.33	0.89
Ni-F	Coarse grain	-0.03(-0.1,0.03)	0.34	0.90
Zn-F	Nuts	-0.04(-0.13,0.04)	0.35	0.91
Li-F	Red meat	-0.06(-0.19,0.07)	0.35	0.91
Zn-F	Dark-colored vegetables	0.03(-0.04,0.11)	0.35	0.91
Ag-F	Nuts	-0.04(-0.11,0.04)	0.35	0.91
Ba-F	Animal offal	0.03(-0.03,0.09)	0.36	0.91
V-F	Legumes and soy products	-0.04(-0.11,0.04)	0.36	0.91
Ni-F	Sugar-sweetened beverages	-0.03(-0.11,0.04)	0.38	0.95
Be-F	Animal offal	0.09(-0.11,0.28)	0.39	0.95
V-F	Processed meat	-0.05(-0.17,0.07)	0.40	0.95
Sn-F	Coffee	0.06(-0.08,0.21)	0.40	0.95
Ga-F	Sugar-sweetened beverages	-0.03(-0.09,0.04)	0.41	0.95
Mo-F	Sugar-sweetened beverages	-0.02(-0.07,0.03)	0.41	0.95
Cd-F	Sugar-sweetened beverages	-0.06(-0.2,0.08)	0.41	0.95
Sn-F	Dark-colored vegetables	0.04(-0.05,0.13)	0.41	0.95
Ga-F	Processed meat	-0.03(-0.1,0.04)	0.41	0.95
Cd-F	Coarse grain	0.05(-0.07,0.18)	0.41	0.95
Al-F	Sugar-sweetened beverages	-0.04(-0.13,0.06)	0.41	0.95
Zn-F	White rice	0.04(-0.05,0.12)	0.42	0.96
Ce-F	Dark-colored vegetables	0.02(-0.03,0.06)	0.43	0.96

Rb-F	Dark-colored vegetables	-0.01(-0.03,0.01)	0.43	0.96
Co-F	Tea	0.03(-0.05,0.12)	0.44	0.96
Cd-F	Fruits	-0.04(-0.15,0.06)	0.44	0.96
Al-F	Nuts	-0.03(-0.11,0.05)	0.45	0.96
Ga-F	Fruits	0.02(-0.03,0.07)	0.46	0.96
Ga-F	Pickled or fried food	0.02(-0.04,0.09)	0.46	0.96
Zn-F	Coffee	0.05(-0.08,0.17)	0.46	0.96
Fe-F	Pickled or fried food	0.02(-0.04,0.09)	0.46	0.96
Co-F	Pickled or fried food	-0.03(-0.12,0.06)	0.47	0.96
Fe-F	Coarse grain	0.02(-0.04,0.08)	0.47	0.96
Sn-F	Animal offal	0.05(-0.08,0.18)	0.47	0.96
Tl-F	Nuts	-0.03(-0.11,0.05)	0.47	0.96
Al-F	Pickled or fried food	-0.03(-0.13,0.06)	0.47	0.96
Fe-F	Processed meat	0.03(-0.05,0.1)	0.47	0.96
Be-F	Pickled or fried food	-0.06(-0.23,0.11)	0.47	0.96
Mo-F	Legumes and soy products	0.01(-0.02,0.05)	0.48	0.96
Ni-F	Animal offal	0.03(-0.06,0.11)	0.50	0.97
Ce-F	Sugar-sweetened beverages	-0.02(-0.08,0.04)	0.50	0.97
Li-F	Legumes and soy products	0.04(-0.09,0.17)	0.50	0.97
Tl-F	Coffee	-0.04(-0.16,0.08)	0.51	0.97
Fe-F	Dark-colored vegetables	-0.02(-0.07,0.03)	0.51	0.97
Cd-F	Processed meat	-0.05(-0.21,0.11)	0.51	0.97
Ag-F	Coffee	0.04(-0.07,0.14)	0.51	0.97
Mo-F	Coffee	-0.02(-0.08,0.04)	0.52	0.97
Ni-F	Fruits	-0.02(-0.08,0.04)	0.53	0.97
Tl-F	Legumes and soy products	-0.02(-0.09,0.05)	0.53	0.97

Co-F	Fruits	0.02(-0.05,0.09)	0.55	0.97
Mo-F	Processed meat	-0.02(-0.08,0.04)	0.56	0.97
Ce-F	Coarse grain	-0.02(-0.07,0.04)	0.56	0.97
Se-F	Sugar-sweetened beverages	-0.01(-0.04,0.02)	0.56	0.97
Fe-F	Tea	0.02(-0.04,0.08)	0.57	0.97
Ag-F	Coarse grain	0.02(-0.05,0.1)	0.57	0.97
Mo-F	Animal offal	-0.02(-0.07,0.04)	0.58	0.97
Mn-F	Red meat	0.02(-0.04,0.08)	0.58	0.97
V-F	Tea	0.03(-0.07,0.13)	0.58	0.97
Sr-F	Coarse grain	0.01(-0.03,0.05)	0.58	0.97
Ga-F	White rice	0.02(-0.04,0.07)	0.59	0.97
Cu-F	Nuts	-0.01(-0.04,0.02)	0.59	0.97
Co-F	Nuts	0.02(-0.06,0.1)	0.59	0.97
Cd-F	Legumes and soy products	0.03(-0.08,0.13)	0.60	0.97
Be-F	Coffee	-0.06(-0.28,0.16)	0.60	0.97
Mo-F	Tea	0.01(-0.03,0.06)	0.60	0.97
Se-F	Fruits	0.01(-0.02,0.03)	0.61	0.97
Mo-F	Red meat	0.01(-0.03,0.05)	0.61	0.97
Li-F	Fruits	0.03(-0.1,0.17)	0.61	0.97
Co-F	Animal offal	0.03(-0.08,0.13)	0.62	0.97
Cu-F	Fruits	0.01(-0.02,0.03)	0.63	0.97
Mn-F	Fruits	-0.02(-0.08,0.05)	0.63	0.97
Mn-F	Legumes and soy products	-0.02(-0.08,0.05)	0.63	0.97
Tl-F	Animal offal	0.03(-0.08,0.13)	0.64	0.97
Ag-F	Tea	-0.02(-0.1,0.06)	0.64	0.97
Be-F	Coarse grain	-0.04(-0.19,0.12)	0.64	0.97

Al-F	Legumes and soy products	-0.02(-0.09,0.05)	0.64	0.97
Fe-F	Fruits	0.01(-0.04,0.06)	0.66	0.97
Fe-F	Legumes and soy products	0.01(-0.04,0.06)	0.67	0.97
Zn-F	Legumes and soy products	-0.02(-0.09,0.06)	0.67	0.97
Tl-F	Tea	-0.02(-0.11,0.07)	0.67	0.97
Zn-F	Red meat	0.02(-0.06,0.09)	0.67	0.97
Be-F	White rice	0.03(-0.13,0.19)	0.68	0.97
Cd-F	Tea	0.03(-0.1,0.16)	0.68	0.97
Zn-F	Sugar-sweetened beverages	-0.02(-0.12,0.08)	0.68	0.97
Al-F	Animal offal	0.02(-0.08,0.13)	0.68	0.97
Li-F	Processed meat	-0.04(-0.24,0.16)	0.68	0.97
Be-F	Legumes and soy products	0.03(-0.1,0.16)	0.68	0.97
Rb-F	Nuts	0.01(-0.02,0.03)	0.69	0.97
Ce-F	Pickled or fried food	0.01(-0.05,0.07)	0.69	0.97
V-F	Dark-colored vegetables	0.02(-0.06,0.1)	0.69	0.97
Be-F	Red meat	-0.03(-0.15,0.1)	0.70	0.97
Ba-F	Legumes and soy products	-0.01(-0.05,0.03)	0.72	0.97
Mn-F	Processed meat	-0.02(-0.11,0.08)	0.72	0.97
Li-F	Coffee	0.04(-0.18,0.26)	0.73	0.97
Sn-F	Nuts	0.02(-0.09,0.12)	0.73	0.97
Se-F	Legumes and soy products	0(-0.02,0.03)	0.73	0.97
Ba-F	Dark-colored vegetables	0.01(-0.04,0.05)	0.73	0.97
Rb-F	Processed meat	0.01(-0.03,0.04)	0.73	0.97
Mo-F	Coarse grain	-0.01(-0.05,0.04)	0.74	0.97
Zn-F	Fruits	-0.01(-0.08,0.06)	0.75	0.97
Li-F	White rice	0.03(-0.13,0.19)	0.75	0.97

Ce-F	Tea	0.01(-0.05,0.06)	0.76	0.97
Li-F	Sugar-sweetened beverages	-0.03(-0.2,0.15)	0.76	0.97
Ce-F	Coffee	-0.01(-0.09,0.06)	0.76	0.97
Se-F	Dark-colored vegetables	0(-0.03,0.02)	0.76	0.97
Ag-F	Dark-colored vegetables	0.01(-0.05,0.08)	0.76	0.97
Rb-F	Pickled or fried food	0(-0.03,0.04)	0.77	0.97
Ba-F	Sugar-sweetened beverages	-0.01(-0.06,0.05)	0.77	0.97
Ba-F	Coarse grain	0.01(-0.04,0.06)	0.78	0.97
Tl-F	Fruits	-0.01(-0.08,0.06)	0.78	0.97
Al-F	White rice	0.01(-0.07,0.1)	0.78	0.97
Mn-F	Animal offal	0.01(-0.08,0.11)	0.78	0.97
Mn-F	Sugar-sweetened beverages	0.01(-0.07,0.1)	0.78	0.97
Mn-F	White rice	-0.01(-0.09,0.07)	0.78	0.97
Ce-F	White rice	0.01(-0.05,0.06)	0.78	0.97
Cu-F	Red meat	0(-0.03,0.02)	0.78	0.97
Tl-F	White rice	-0.01(-0.1,0.07)	0.79	0.97
Be-F	Fruits	-0.02(-0.15,0.11)	0.79	0.97
Co-F	White rice	0.01(-0.07,0.09)	0.80	0.97
Co-F	Red meat	-0.01(-0.08,0.06)	0.80	0.97
Se-F	Coffee	0(-0.03,0.04)	0.80	0.97
Rb-F	Sugar-sweetened beverages	0(-0.03,0.04)	0.81	0.97
Cd-F	Dark-colored vegetables	0.01(-0.09,0.12)	0.82	0.97
Se-F	Animal offal	0(-0.03,0.04)	0.83	0.97
Mo-F	Pickled or fried food	0.01(-0.04,0.06)	0.83	0.97
Rb-F	White rice	0(-0.03,0.03)	0.83	0.97
Cd-F	Coffee	0.02(-0.16,0.2)	0.83	0.97

Ba-F	Processed meat	0.01(-0.06,0.07)	0.83	0.97
Al-F	Processed meat	0.01(-0.1,0.12)	0.83	0.97
Sr-F	Nuts	0(-0.03,0.04)	0.84	0.97
Zn-F	Processed meat	0.01(-0.1,0.12)	0.85	0.97
Cu-F	Legumes and soy products	0(-0.03,0.02)	0.85	0.97
Se-F	Tea	0(-0.03,0.03)	0.85	0.97
Fe-F	Red meat	0(-0.04,0.05)	0.85	0.97
Ce-F	Legumes and soy products	0(-0.04,0.05)	0.85	0.97
Fe-F	Coffee	0.01(-0.07,0.09)	0.85	0.97
Se-F	Pickled or fried food	0(-0.03,0.03)	0.85	0.97
Ga-F	Legumes and soy products	0(-0.04,0.05)	0.85	0.97
Sn-F	Processed meat	0.01(-0.12,0.15)	0.85	0.97
Se-F	Processed meat	0(-0.04,0.03)	0.86	0.97
Cd-F	Animal offal	0.01(-0.14,0.17)	0.86	0.97
V-F	Coffee	0.01(-0.12,0.14)	0.86	0.97
Ga-F	Nuts	0(-0.06,0.05)	0.87	0.97
Fe-F	Nuts	0(-0.05,0.06)	0.87	0.97
Ce-F	Nuts	0(-0.05,0.06)	0.88	0.97
Ga-F	Coffee	-0.01(-0.09,0.07)	0.88	0.97
Se-F	White rice	0(-0.03,0.03)	0.88	0.97
Cu-F	Dark-colored vegetables	0(-0.03,0.03)	0.89	0.97
Li-F	Pickled or fried food	0.01(-0.16,0.19)	0.89	0.97
Ni-F	Nuts	0(-0.06,0.07)	0.89	0.97
Be-F	Dark-colored vegetables	-0.01(-0.14,0.12)	0.89	0.97
Ba-F	Fruits	0(-0.04,0.04)	0.89	0.97
Mn-F	Pickled or fried food	0.01(-0.08,0.09)	0.90	0.97

Tl-F	Coarse grain	-0.01(-0.09,0.08)	0.90	0.97
Co-F	Processed meat	-0.01(-0.11,0.1)	0.91	0.97
Ag-F	Sugar-sweetened beverages	0(-0.08,0.09)	0.91	0.97
Fe-F	Animal offal	0(-0.07,0.08)	0.91	0.97
Sr-F	Legumes and soy products	0(-0.03,0.03)	0.91	0.97
Ni-F	Processed meat	0(-0.08,0.09)	0.92	0.97
Ba-F	Nuts	0(-0.05,0.05)	0.92	0.97
Rb-F	Legumes and soy products	0(-0.02,0.02)	0.92	0.97
Be-F	Tea	0.01(-0.15,0.17)	0.92	0.97
Cd-F	Nuts	0.01(-0.12,0.13)	0.92	0.97
Rb-F	Animal offal	0(-0.04,0.03)	0.93	0.97
Ag-F	Animal offal	0(-0.1,0.09)	0.93	0.97
Co-F	Legumes and soy products	0(-0.06,0.07)	0.93	0.97
Co-F	Sugar-sweetened beverages	0(-0.09,0.1)	0.94	0.97
Be-F	Processed meat	-0.01(-0.2,0.19)	0.95	0.98
Sr-F	Sugar-sweetened beverages	0(-0.04,0.05)	0.96	0.98
Cu-F	Tea	0(-0.03,0.03)	0.96	0.98
Li-F	Nuts	0(-0.15,0.16)	0.96	0.98
V-F	White rice	0(-0.1,0.09)	0.97	0.98
Ni-F	Dark-colored vegetables	0(-0.06,0.06)	0.98	0.99
Ni-F	Legumes and soy products	0(-0.06,0.06)	0.99	1.00
Ni-F	Red meat	0(-0.06,0.06)	0.99	1.00
Mo-F	Dark-colored vegetables	0(-0.04,0.04)	1.00	1.00

Table S9. Association between dietary intake and trace elements concentrations among 1,066 males.

Trace elements-Males	Dietary	Beta (95% CI)	P-value	P-FDR
Rb-M	Tea	13.13(6.87,19.38)	0.00	0.01
Ag-M	Coffee	0.08(0.04,0.12)	0.00	0.02
Ag-M	Processed meat	0.06(0.03,0.09)	0.00	0.05
Co-M	Coarse grain	0.04(0.02,0.07)	0.00	0.08
Zn-M	Nuts	2.11(0.76,3.45)	0.00	0.13
Ag-M	Pickled or fried food	0.04(0.01,0.07)	0.00	0.19
Ag-M	Sugar-sweetened beverages	0.04(0.01,0.07)	0.01	0.23
V-M	Processed meat	0.12(0.03,0.2)	0.01	0.23
Co-M	Nuts	0.04(0.01,0.06)	0.01	0.23
Ag-M	Coarse grain	0.04(0.01,0.07)	0.01	0.28
V-M	Coffee	0.13(0.03,0.24)	0.01	0.28
V-M	Sugar-sweetened beverages	0.09(0.02,0.16)	0.01	0.28
Al-M	Coffee	12.02(2.51,21.54)	0.01	0.28
Zn-M	Sugar-sweetened beverages	1.61(0.3,2.92)	0.02	0.31
V-M	Nuts	0.09(0.02,0.16)	0.02	0.31
V-M	Pickled or fried food	0.09(0.01,0.16)	0.02	0.31
Zn-M	Coffee	2.25(0.36,4.15)	0.02	0.31
Zn-M	Processed meat	1.85(0.29,3.42)	0.02	0.31
Zn-M	Coarse grain	1.59(0.24,2.94)	0.02	0.31
Se-M	Red meat	2.46(0.21,4.71)	0.03	0.39
Zn-M	Pickled or fried food	1.47(0.12,2.82)	0.03	0.39
Al-M	Animal offal	8.41(0.61,16.21)	0.03	0.39

Ga-M	Legumes and soy products	0.03(0,0.06)	0.04	0.39
Mo-M	White rice	2.22(0.14,4.31)	0.04	0.39
Ce-M	Fruits	8.36(0.52,16.2)	0.04	0.39
Ga-M	Fruits	0.03(0,0.06)	0.04	0.39
Mo-M	Coffee	2.55(0.12,4.99)	0.04	0.40
V-M	Coarse grain	0.08(0,0.15)	0.04	0.40
Rb-M	Processed meat	-8.82(-17.68,0.03)	0.05	0.48
Mo-M	Legumes and soy products	1.36(-0.01,2.74)	0.05	0.48
Sr-M	Coffee	2.08(-0.09,4.24)	0.06	0.54
Se-M	Sugar-sweetened beverages	-2.34(-4.82,0.13)	0.06	0.54
Al-M	Fruits	5.01(-0.32,10.34)	0.07	0.55
Al-M	Legumes and soy products	5(-0.39,10.38)	0.07	0.56
Sr-M	Tea	1.17(-0.11,2.44)	0.07	0.57
Cd-M	Red meat	0.02(0,0.05)	0.07	0.58
Tl-M	Tea	-0.02(-0.04,0)	0.08	0.59
Be-M	Tea	0.02(0,0.04)	0.08	0.59
Li-M	Coarse grain	9.12(-1.18,19.43)	0.08	0.59
V-M	White rice	0.08(-0.01,0.17)	0.09	0.59
Zn-M	Animal offal	1.36(-0.2,2.91)	0.09	0.59
Tl-M	Red meat	-0.02(-0.04,0)	0.09	0.59
Al-M	Processed meat	6.76(-1.11,14.64)	0.09	0.60
Ni-M	Coarse grain	0.11(-0.02,0.23)	0.10	0.64
V-M	Animal offal	0.07(-0.02,0.15)	0.11	0.66
Zn-M	Fruits	0.87(-0.2,1.93)	0.11	0.66
V-M	Tea	0.05(-0.01,0.11)	0.11	0.66
Sr-M	Animal offal	1.42(-0.35,3.2)	0.12	0.66

Al-M	Pickled or fried food	5.42(-1.35,12.18)	0.12	0.66
Ga-M	Tea	0.02(-0.01,0.06)	0.12	0.66
V-M	Fruits	0.05(-0.01,0.1)	0.12	0.66
Sr-M	Nuts	1.22(-0.32,2.76)	0.12	0.66
Mn-M	Tea	0.21(-0.06,0.48)	0.12	0.66
Ba-M	White rice	-3.04(-6.97,0.89)	0.13	0.68
Sn-M	Nuts	-0.84(-1.94,0.26)	0.13	0.69
Zn-M	Tea	0.84(-0.27,1.96)	0.14	0.70
Be-M	Sugar-sweetened beverages	-0.02(-0.04,0.01)	0.14	0.70
Li-M	White rice	-9.02(-21.44,3.41)	0.15	0.76
Sr-M	Red meat	0.97(-0.39,2.33)	0.16	0.78
Tl-M	Dark-colored vegetables	-0.01(-0.04,0.01)	0.17	0.79
Zn-M	Red meat	0.84(-0.36,2.03)	0.17	0.79
Ag-M	Legumes and soy products	0.02(-0.01,0.04)	0.18	0.81
Mo-M	Tea	-0.97(-2.4,0.46)	0.18	0.82
Ce-M	Pickled or fried food	6.77(-3.2,16.74)	0.18	0.82
Ce-M	Coarse grain	-6.59(-16.58,3.39)	0.20	0.82
Fe-M	Tea	0.13(-0.07,0.32)	0.20	0.82
Li-M	Red meat	5.9(-3.22,15.01)	0.20	0.82
Ba-M	Fruits	1.65(-0.92,4.21)	0.21	0.82
Rb-M	Animal offal	-5.63(-14.42,3.15)	0.21	0.82
Ce-M	Legumes and soy products	5.06(-2.88,13.01)	0.21	0.82
Sn-M	Legumes and soy products	0.55(-0.32,1.43)	0.21	0.82
Ba-M	Coffee	2.88(-1.71,7.47)	0.22	0.82
Mn-M	Legumes and soy products	0.16(-0.1,0.42)	0.22	0.82
Al-M	Nuts	4.16(-2.62,10.94)	0.23	0.82

Tl-M	Nuts	-0.02(-0.04,0.01)	0.23	0.82
Zn-M	White rice	0.99(-0.64,2.62)	0.23	0.82
Mo-M	Pickled or fried food	1.04(-0.69,2.77)	0.24	0.82
Cu-M	Fruits	-0.04(-0.11,0.03)	0.24	0.82
Ba-M	Legumes and soy products	1.55(-1.05,4.14)	0.24	0.82
Ag-M	Tea	0.01(-0.01,0.04)	0.24	0.82
Cu-M	Nuts	-0.05(-0.15,0.04)	0.25	0.82
Tl-M	White rice	-0.02(-0.05,0.01)	0.25	0.82
V-M	Legumes and soy products	0.03(-0.02,0.09)	0.25	0.82
Li-M	Tea	4.96(-3.57,13.48)	0.25	0.82
V-M	Red meat	0.04(-0.03,0.1)	0.25	0.82
Cu-M	Red meat	0.05(-0.03,0.13)	0.26	0.82
Sr-M	Fruits	-0.68(-1.89,0.53)	0.27	0.84
Rb-M	White rice	5.09(-4.1,14.28)	0.28	0.84
Sr-M	Legumes and soy products	0.67(-0.55,1.9)	0.28	0.84
Be-M	Legumes and soy products	0.01(-0.01,0.03)	0.28	0.84
Co-M	Red meat	-0.01(-0.04,0.01)	0.28	0.84
Ga-M	Coffee	0.03(-0.02,0.08)	0.29	0.85
Ga-M	Pickled or fried food	0.02(-0.02,0.06)	0.29	0.85
Sr-M	Pickled or fried food	0.82(-0.72,2.36)	0.30	0.85
Se-M	Dark-colored vegetables	1.12(-1,3.25)	0.30	0.85
Sr-M	Dark-colored vegetables	0.68(-0.61,1.97)	0.30	0.85
Se-M	Processed meat	-1.55(-4.51,1.41)	0.31	0.85
Rb-M	Coffee	5.52(-5.22,16.25)	0.31	0.86
Al-M	Sugar-sweetened beverages	3.33(-3.26,9.92)	0.32	0.87
Se-M	Tea	1.05(-1.06,3.15)	0.33	0.88

Rb-M	Dark-colored vegetables	-3.13(-9.5,3.23)	0.33	0.88
Fe-M	Legumes and soy products	0.09(-0.1,0.28)	0.34	0.88
Mn-M	Dark-colored vegetables	-0.13(-0.4,0.14)	0.34	0.88
Fe-M	White rice	-0.14(-0.43,0.15)	0.34	0.88
Rb-M	Nuts	-3.66(-11.29,3.96)	0.35	0.88
Mn-M	Red meat	0.14(-0.15,0.43)	0.35	0.88
Fe-M	Coffee	0.16(-0.18,0.49)	0.35	0.88
Se-M	Nuts	-1.19(-3.74,1.35)	0.36	0.88
Ni-M	White rice	0.07(-0.08,0.22)	0.36	0.88
Al-M	Coarse grain	3.14(-3.64,9.92)	0.36	0.88
Ga-M	Processed meat	0.02(-0.02,0.06)	0.36	0.88
Mo-M	Dark-colored vegetables	-0.66(-2.11,0.78)	0.37	0.88
Li-M	Fruits	-3.7(-11.81,4.42)	0.37	0.88
Ni-M	Dark-colored vegetables	-0.05(-0.15,0.06)	0.37	0.88
Se-M	White rice	1.38(-1.69,4.45)	0.38	0.88
Ni-M	Fruits	-0.04(-0.14,0.05)	0.38	0.88
Tl-M	Fruits	-0.01(-0.03,0.01)	0.38	0.88
Ce-M	Nuts	-4.43(-14.42,5.56)	0.38	0.88
Cu-M	Coffee	0.06(-0.07,0.18)	0.39	0.88
Cu-M	Dark-colored vegetables	-0.03(-0.11,0.04)	0.39	0.88
Se-M	Animal offal	1.26(-1.68,4.19)	0.40	0.88
Tl-M	Legumes and soy products	0.01(-0.01,0.03)	0.40	0.88
Ce-M	White rice	-5.14(-17.18,6.89)	0.40	0.88
Sr-M	Processed meat	0.76(-1.03,2.55)	0.40	0.88
Al-M	Red meat	2.53(-3.47,8.52)	0.41	0.88
Be-M	White rice	0.01(-0.02,0.04)	0.41	0.88

Ce-M	Sugar-sweetened beverages	-4.05(-13.76,5.65)	0.41	0.88
Li-M	Legumes and soy products	-3.33(-11.54,4.87)	0.43	0.90
Ba-M	Pickled or fried food	1.3(-1.96,4.56)	0.43	0.90
Ga-M	Sugar-sweetened beverages	-0.01(-0.05,0.02)	0.44	0.90
Co-M	Pickled or fried food	0.01(-0.02,0.04)	0.46	0.92
Sn-M	Coarse grain	0.42(-0.68,1.52)	0.46	0.92
Be-M	Processed meat	0.01(-0.02,0.04)	0.46	0.92
Co-M	Dark-colored vegetables	-0.01(-0.03,0.01)	0.46	0.92
Ni-M	Red meat	-0.04(-0.15,0.07)	0.46	0.92
Ag-M	Fruits	0.01(-0.01,0.03)	0.46	0.92
Sr-M	Coarse grain	0.57(-0.97,2.11)	0.47	0.92
Ga-M	Animal offal	0.02(-0.03,0.06)	0.47	0.92
Li-M	Dark-colored vegetables	3.11(-5.5,11.72)	0.48	0.92
Ba-M	Red meat	-1.02(-3.9,1.87)	0.49	0.92
Ce-M	Animal offal	4.05(-7.46,15.56)	0.49	0.92
Ga-M	Coarse grain	-0.01(-0.05,0.02)	0.49	0.92
Cd-M	White rice	0.01(-0.02,0.04)	0.49	0.92
Zn-M	Legumes and soy products	0.38(-0.7,1.45)	0.49	0.92
V-M	Dark-colored vegetables	-0.02(-0.08,0.04)	0.51	0.93
Sn-M	Animal offal	-0.42(-1.69,0.85)	0.51	0.93
Se-M	Coarse grain	-0.85(-3.4,1.7)	0.51	0.93
Co-M	Coffee	0.01(-0.03,0.05)	0.52	0.93
Ba-M	Nuts	-1.06(-4.33,2.2)	0.52	0.93
Se-M	Pickled or fried food	-0.83(-3.37,1.72)	0.52	0.93
Ag-M	Animal offal	0.01(-0.02,0.04)	0.53	0.93
Cd-M	Animal offal	0.01(-0.02,0.04)	0.53	0.93

Cd-M	Legumes and soy products	-0.01(-0.03,0.01)	0.54	0.93
Fe-M	Sugar-sweetened beverages	-0.07(-0.3,0.16)	0.54	0.93
Li-M	Animal offal	-3.7(-15.59,8.2)	0.54	0.93
Ga-M	White rice	-0.01(-0.06,0.03)	0.54	0.93
Cd-M	Pickled or fried food	0.01(-0.02,0.04)	0.54	0.93
Be-M	Nuts	-0.01(-0.03,0.02)	0.56	0.93
Ni-M	Tea	0.03(-0.07,0.13)	0.56	0.93
Se-M	Fruits	0.59(-1.42,2.59)	0.56	0.93
Li-M	Coffee	-4.26(-18.79,10.26)	0.56	0.93
Ba-M	Animal offal	1.1(-2.66,4.86)	0.57	0.93
Co-M	Tea	0.01(-0.02,0.03)	0.57	0.93
Cu-M	Tea	0.02(-0.05,0.1)	0.57	0.93
Ga-M	Red meat	-0.01(-0.04,0.02)	0.58	0.93
Fe-M	Coarse grain	-0.07(-0.31,0.17)	0.58	0.93
Fe-M	Processed meat	0.08(-0.2,0.35)	0.58	0.93
Sn-M	Dark-colored vegetables	-0.26(-1.18,0.66)	0.58	0.93
Ce-M	Tea	2.32(-5.94,10.58)	0.58	0.93
Cu-M	Sugar-sweetened beverages	0.02(-0.06,0.11)	0.58	0.93
Ce-M	Coffee	3.86(-10.2,17.92)	0.59	0.93
Be-M	Dark-colored vegetables	-0.01(-0.03,0.02)	0.60	0.93
Ba-M	Coarse grain	-0.87(-4.13,2.4)	0.60	0.93
Li-M	Sugar-sweetened beverages	-2.66(-12.69,7.37)	0.60	0.93
Li-M	Processed meat	-3.1(-15.1,8.89)	0.61	0.93
Be-M	Red meat	-0.01(-0.03,0.02)	0.62	0.93
Fe-M	Fruits	-0.05(-0.24,0.14)	0.62	0.93
Sn-M	Processed meat	-0.32(-1.61,0.96)	0.62	0.93

Sr-M	White rice	-0.46(-2.32,1.39)	0.62	0.93
Ga-M	Nuts	-0.01(-0.05,0.03)	0.63	0.93
Fe-M	Pickled or fried food	0.06(-0.18,0.3)	0.63	0.93
Fe-M	Nuts	-0.06(-0.3,0.18)	0.63	0.93
Fe-M	Red meat	0.05(-0.16,0.26)	0.64	0.93
Al-M	White rice	-1.91(-10.08,6.26)	0.65	0.93
Mo-M	Coarse grain	-0.4(-2.14,1.33)	0.65	0.93
Cd-M	Nuts	-0.01(-0.03,0.02)	0.65	0.93
Mn-M	Coffee	0.11(-0.35,0.56)	0.65	0.93
Fe-M	Animal offal	-0.06(-0.34,0.21)	0.66	0.93
Ba-M	Sugar-sweetened beverages	-0.71(-3.88,2.47)	0.66	0.93
Li-M	Pickled or fried food	-2.27(-12.57,8.04)	0.67	0.93
Ni-M	Nuts	0.03(-0.1,0.15)	0.67	0.93
Be-M	Fruits	0(-0.02,0.02)	0.67	0.93
Tl-M	Coffee	0.01(-0.03,0.04)	0.67	0.93
Mo-M	Animal offal	-0.42(-2.41,1.58)	0.68	0.93
Tl-M	Sugar-sweetened beverages	0.01(-0.02,0.03)	0.68	0.93
Rb-M	Red meat	1.37(-5.37,8.12)	0.69	0.93
Co-M	White rice	0.01(-0.03,0.04)	0.69	0.93
Mo-M	Fruits	-0.27(-1.64,1.09)	0.69	0.93
Ni-M	Legumes and soy products	-0.02(-0.12,0.08)	0.70	0.93
Cu-M	Animal offal	0.02(-0.08,0.13)	0.70	0.93
Se-M	Legumes and soy products	-0.39(-2.42,1.63)	0.70	0.93
Cu-M	Legumes and soy products	0.01(-0.06,0.09)	0.71	0.93
Ag-M	Red meat	0(-0.03,0.02)	0.71	0.93
Sn-M	Sugar-sweetened beverages	0.2(-0.87,1.27)	0.72	0.93

Li-M	Nuts	-1.89(-12.22,8.43)	0.72	0.93
Cu-M	Coarse grain	0.02(-0.07,0.11)	0.72	0.93
Cd-M	Dark-colored vegetables	0(-0.03,0.02)	0.72	0.93
Mn-M	White rice	0.07(-0.32,0.46)	0.72	0.93
Ba-M	Tea	0.48(-2.22,3.17)	0.73	0.93
Cu-M	Processed meat	0.02(-0.09,0.12)	0.73	0.93
Ag-M	White rice	0.01(-0.03,0.04)	0.74	0.93
Tl-M	Animal offal	0(-0.02,0.03)	0.74	0.93
Mo-M	Red meat	0.26(-1.27,1.79)	0.74	0.93
Cd-M	Sugar-sweetened beverages	0(-0.02,0.03)	0.75	0.93
Rb-M	Legumes and soy products	-1(-7.06,5.07)	0.75	0.93
Zn-M	Dark-colored vegetables	-0.18(-1.31,0.94)	0.75	0.93
Ba-M	Processed meat	0.61(-3.18,4.41)	0.75	0.93
Cu-M	White rice	0.02(-0.09,0.13)	0.75	0.93
Ga-M	Dark-colored vegetables	0(-0.04,0.03)	0.75	0.93
Sn-M	Pickled or fried food	-0.17(-1.28,0.93)	0.76	0.93
Rb-M	Pickled or fried food	-1.2(-8.82,6.42)	0.76	0.93
Sn-M	White rice	0.2(-1.12,1.53)	0.76	0.93
Tl-M	Coarse grain	0(-0.02,0.03)	0.76	0.93
Tl-M	Pickled or fried food	0(-0.03,0.02)	0.77	0.93
Co-M	Processed meat	0(-0.03,0.04)	0.77	0.93
Mn-M	Processed meat	0.05(-0.33,0.43)	0.78	0.93
Mn-M	Fruits	-0.04(-0.29,0.22)	0.79	0.93
Cu-M	Pickled or fried food	0.01(-0.08,0.1)	0.79	0.93
Cd-M	Coarse grain	0(-0.03,0.02)	0.79	0.93
Co-M	Legumes and soy products	0(-0.02,0.02)	0.80	0.94

Rb-M	Fruits	0.71(-5.29,6.72)	0.82	0.95
Ag-M	Nuts	0(-0.03,0.03)	0.82	0.95
Al-M	Dark-colored vegetables	0.65(-5.01,6.31)	0.82	0.95
Ce-M	Processed meat	1.16(-10.46,12.77)	0.85	0.96
Mn-M	Animal offal	0.04(-0.34,0.41)	0.85	0.96
Mn-M	Coarse grain	0.03(-0.29,0.36)	0.85	0.96
Se-M	Coffee	0.35(-3.24,3.93)	0.85	0.96
Ba-M	Dark-colored vegetables	0.26(-2.46,2.98)	0.85	0.96
Ni-M	Pickled or fried food	0.01(-0.11,0.14)	0.85	0.96
Mn-M	Sugar-sweetened beverages	0.03(-0.29,0.35)	0.85	0.96
Cd-M	Tea	0(-0.02,0.02)	0.86	0.96
Mo-M	Processed meat	0.18(-1.83,2.19)	0.86	0.96
Ni-M	Animal offal	0.01(-0.13,0.16)	0.86	0.96
Sn-M	Tea	0.08(-0.83,0.99)	0.86	0.96
Rb-M	Sugar-sweetened beverages	-0.54(-7.95,6.88)	0.89	0.98
Ce-M	Dark-colored vegetables	-0.57(-8.91,7.77)	0.89	0.98
Tl-M	Processed meat	0(-0.03,0.03)	0.89	0.98
Ce-M	Red meat	-0.58(-9.41,8.25)	0.90	0.98
Ni-M	Sugar-sweetened beverages	0.01(-0.11,0.13)	0.90	0.98
Be-M	Pickled or fried food	0(-0.03,0.02)	0.91	0.98
Ni-M	Coffee	-0.01(-0.19,0.17)	0.91	0.98
Ag-M	Dark-colored vegetables	0(-0.03,0.02)	0.92	0.98
Rb-M	Coarse grain	0.4(-7.23,8.03)	0.92	0.98
Ni-M	Processed meat	0.01(-0.14,0.15)	0.92	0.98
Be-M	Coarse grain	0(-0.02,0.03)	0.93	0.98
Co-M	Animal offal	0(-0.03,0.03)	0.93	0.98

Co-M	Fruits	0(-0.02,0.02)	0.93	0.98
Mo-M	Sugar-sweetened beverages	0.07(-1.61,1.76)	0.93	0.98
Mo-M	Nuts	-0.07(-1.81,1.66)	0.93	0.98
Sn-M	Fruits	-0.03(-0.9,0.83)	0.94	0.98
Cd-M	Coffee	0(-0.04,0.04)	0.94	0.98
Mn-M	Nuts	0.01(-0.32,0.34)	0.95	0.98
Al-M	Tea	-0.18(-5.78,5.43)	0.95	0.98
Sr-M	Sugar-sweetened beverages	-0.04(-1.54,1.45)	0.95	0.99
Be-M	Coffee	0(-0.04,0.04)	0.97	0.99
Sn-M	Coffee	-0.03(-1.58,1.52)	0.97	0.99
Co-M	Sugar-sweetened beverages	0(-0.03,0.03)	0.97	0.99
Be-M	Animal offal	0(-0.03,0.03)	0.98	0.99
Sn-M	Red meat	-0.01(-0.99,0.96)	0.98	0.99
Mn-M	Pickled or fried food	0(-0.33,0.32)	0.98	0.99
Fe-M	Dark-colored vegetables	0(-0.2,0.2)	1.00	1.00
Cd-M	Fruits	0(-0.02,0.02)	1.00	1.00
Cd-M	Processed meat	0(-0.03,0.03)	1.00	1.00
